# SKP2 inactivation suppresses prostate tumorigenesis by mediating JARID1B ubiquitination

**DOI:** 10.18632/oncotarget.2718

**Published:** 2014-12-23

**Authors:** Wenfu Lu, Shenji Liu, Bo Li, Yingqiu Xie, Christine Adhiambo, Qing Yang, Billy R. Ballard, Keiichi I. Nakayama, Robert J. Matusik, Zhenbang Chen

**Affiliations:** ^1^ Department of Biochemistry and Cancer Biology, Meharry Medical College, TN 37208, USA; ^2^ Department of Pathology, Anatomy and Cell Biology, Meharry Medical College, TN 37208, USA; ^3^ Department of Molecular and Cellular Biology, Medical Institute of Bioregulation, Kyushu University, Fukuoka 812-8582, Japan; ^4^ Department of Urologic Surgery, Vanderbilt University School of Medicine, TN 37232, USA

**Keywords:** PTEN, SKP2, JARID1B, TRAF6, histone modifications, prostate cancer

## Abstract

Aberrant elevation of JARID1B and histone H3 lysine 4 trimethylation (H3K4me3) is frequently observed in many diseases including prostate cancer (PCa), yet the mechanisms on the regulation of JARID1B and H3K4me3 through epigenetic alterations still remain poorly understood. Here we report that Skp2 modulates JARID1B and H3K4me3 levels *in vitro* in cultured cells and *in vivo* in mouse models. We demonstrated that Skp2 inactivation decreased H3K4me3 levels, along with a reduction of cell growth, cell migration and malignant transformation of Pten/Trp53 double null MEFs, and further restrained prostate tumorigenesis of Pten/Trp53 mutant mice. Mechanistically, Skp2 decreased the K63-linked ubiquitination of JARID1B by E3 ubiquitin ligase TRAF6, thus decreasing JARID1B demethylase activity and in turn increasing H3K4me3. In agreement, Skp2 deficiency resulted in an increase of JARID1B ubiquitination and in turn a reduction of H3K4me3, and induced senescence through JARID1B accumulation in nucleoli of PCa cells and prostate tumors of mice. Furthermore, we showed that the elevations of Skp2 and H3K4me3 contributed to castration-resistant prostate cancer (CRPC) in mice, and were positively correlated in human PCa specimens. Taken together, our findings reveal a novel network of SKP2- JARID1B, and targeting SKP2 and JARID1B may be a potential strategy for PCa control.

## INTRODUCTION

Prostate cancer (PCa) is the second leading cause of cancer-related deaths in American males [1]. Molecular mechanisms leading to this malignancy are complicate but need to be elucidated in order to develop efficient pathways-targeted chemotherapies. SKP2 (S-phase kinase associated protein-2) is an E3 ligase and an F-box protein component of SKP2 SCF complex (Skp1-Cul1-F-Box) to trigger the ubiquitin-mediated degradation of p27 and other proteins [2, 3]. As a proto-oncogene, SKP2 overexpression is frequently observed in various human cancers including PCa [4, 5], and aberrant elevation of SKP2 is associated with poor prognosis of cancers [6, 7]. Studies demonstrated that SKP2 has oncogenic impact on the initiation and progression of PCa, which correlating with castration resistant prostate cancer (CRPC). Emerging evidence revealed that SKP2 plays an essential role in cell cycle proliferation, cellular senescence, cancer progression, and metastasis [6, 8–10]. This phenomenon is likely a result of degrading the downstream substrates in cell cycles such as p27 [8, 11]. SKP2 regulates a variety of downstream targets hence alters a broad spectrum of signaling cascades [11]. However, SKP2 affects the expression of proteins other than its substrates such as ATF4 [8], RhoA and Miz1 [10] and C-Myc [12, 13], suggesting its essential roles independent of E3 ligase in PCa. Recent studies indicated that SKP2 has novel functions in glycolysis and tumorigenesis through the regulation of AKT ubiquitination in human breast cancer [14]. Moreover, SKP2 is reported to affect the levels of androgen receptor via ubiquitination in PCa [15]. Despite these advancements, the role of SKP2 on the regulation of histone modifications in human PCa still remains elusive.

Global histone modifications including lysine methylation and acetylation have been reported to correlate with human cancers [16–18]. Histone methylation is a dynamic biochemical process that is tightly controlled by histone methyltransferases and histone demethylases to balance biological functions in cells. Deregulation of these epigenetic modification enzymes contributes to the development and progression of PCa through the activation or suppression of gene functions [17–20]. Recent reports show that the increase of JARID1B/KDM5B/PLU1, a specific histone demethylase for H3K4 trimethylation and dimethylation, is frequently observed in PCa specimens, implicating its potential oncogenic roles in PCa [17–20]. The deregulation of trimethylation of lysine 4 on histone H3 (H3K4me3), is contributing to the development of primary PCa as well as progression to CRPC [18]. In addition, aberrant elevation of H3K4me3 also contributes to an epigenetic switch of a number of oncogenes and tumor suppressor genes in PCa cells and furthermore to cancer stem cells [19, 21]. However, the mechanisms underlying the deregulation of H3K4me3 by JARID1B and the relevance with SKP2 in cancers are poorly understood.

Substantial evidence indicates SKP2 plays a critical role in tumorigenesis by regulating a variety of downstream effectors of cellular progresses [6, 8–11, 14]. For example, SKP2 is reported to contribute to the epigenetic silencing of DAB2IP in PCa [22]. Although the role of histone methyltransferase MLL in PCa is unclear, it was reported that leukemogenic MLL fusions were resistant to SKP2-mediated degradation, suggesting the involvement of SKP2 in epigenetic modification mechanisms of histone in Leukemia [23]. Among various histone modifications, H3K4me3 is significantly increased in both localized and hormone refractory PCa [17, 24]. Therefore, we reasoned that SKP2 may be involved in the epigenetic events including histone methylation modifications by regulating histone methylation/demethylation enzymes in PCa.

In this study, we discovered that the aberrant levels of H3K4me3 are remarkably reduced by SKP2 ablation in PCa cells and tumors. We demonstrated that SKP2 determines H3K4me3 levels through inhibiting the K63-linked ubiquitination of JARID1B by E3 ubiquitin ligase TRAF6, resulting in repression of JARID1B demethylase activity. Our findings on histone modifications highlight the implications of a combinatorial targeting on SKP2 and JARID1B for PCa treatment.

## RESULTS

### Skp2 deficiency restrains the prostate tumor growth of *Pten/Trp53* mutant mice

To explore the role of SKP2 on epigenetics and the relevance on PCa progression *in vivo*, we wished to investigate whether Skp2 deficiency suppresses prostate tumorigenesis through affecting the functional coupling of JARID1B and H3K4me3 in mouse models. We focused on JARID1B as it controls H3K4me3 and its abnormal upregulation is frequently observed in PCa [17–20]. To test this possibility, we took advantage of *Pten/Trp53* mouse model to generate *Pten/Trp53/Skp2* conditional triple null (*Pten^pc−/−^*;Trp53*^pc−/−^*;Skp2*^−/−^*)** mutant mice, and subsequently assessed their prostate tumorigenesis. In agreement with previous report [25], *Pten/Trp53* conditional double null (*Pten^pc−/−^*; Trp53*^pc−/−^*) mice developed prostate tumors at 3 months of age, and the average weight of anterior prostates (AP) was 5-fold heavier than that of the age-matched wild type (*WT*) cohort (Supplementary Figure S1A and S1B). The enlargement of AP in *Pten^pc−/−^*;Trp53*^pc−/−^* mice was noticeable when dissected, and marked pathological changes including high-grade prostatic intraepithelial neoplasia (HG-PIN) and invasive cancer were observed in all mice (Supplementary Figure S1C). Importantly, Skp2 deficiency resulted in a suppression of development of prostate tumorigenesis in *Pten^pc−/−^*; Trp53*^pc−/−^* mice, while Skp2 null alone did not cause morphological changes of prostates. The average AP weight of *Pten^pc−/−^*; Trp53*^pc−/−^*; Skp2**^−/−^ mice reduced about 50% as compared to that of *Pten^pc−/−^*; Trp53*^pc−/−^* mice at 3 months of age (*P* < 0.05, Supplementary Figure S1A and S1B). Prostate tumors in *Pten^pc−/−^*; Trp53*^pc−/−^* mice developed microinvasion with cells in atypical nucleus, while age-matched *Pten^pc−/−^*; Trp53*^pc−/−^*; Skp2**^−/−^ mice showed the less severe abnormality of prostate glands with PIN lesions (Supplementary Figure S1C). These data indicate that Skp2 deficiency reduces the development and progression of PCa *in vivo*.

**Figure 1:**
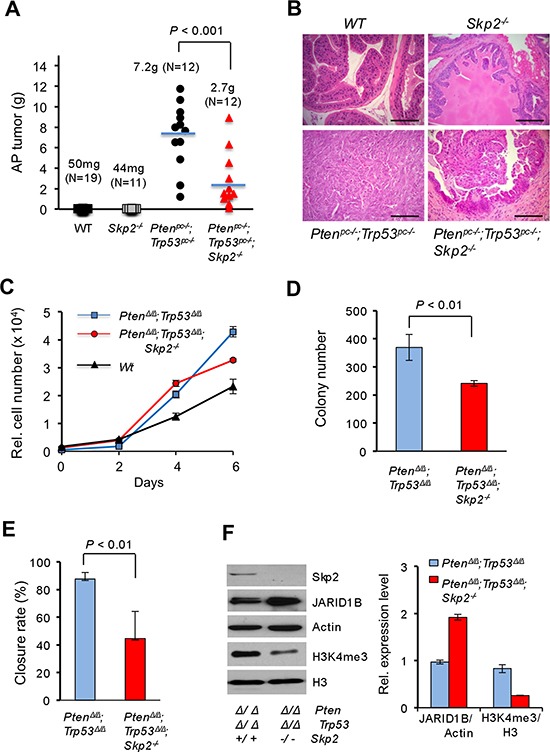
Skp2 inactivation suppresses prostate cancer progression *in vivo* in mice and cell growth of MEF by regulating JARID1B and H3K4me3 *in vitro* **(A)** Quantification analysis of average weights of anterior prostate (AP) tumors of mice at 5–6 months of age. The number of mice for each group is as indicated. The representative biopsies of AP from indicated genotypes of mice are shown in Supplementary Figure S1D. **(B)** H&E staining of prostate tissues from indicated genotypes of mice at 5–6 months of age. Scale bars represent 100 μm. **(C)** Effects of Skp2 inactivation on the cell proliferation of Pten/Trp53 null MEFs. **(D)** Effects of Skp2 inactivation on soft agar transformation of Pten/Trp53 null MEFs. **(E)** Wound healing (migration) assay of Pten/Trp53 null MEFs upon SKP2 inactivation (Also see Supplementary Figure S1E). **(F)** Left panel, Western blot analysis of protein levels of JARID1B and H3K4me3 upon Skp2 inactivation in Pten/Trp53 null MEFs. Right panel, quantification analysis of protein levels for JARID1B and H3K4me3 in MEFs upon Skp2 inactivation. Error bars for C-F represent means ±SD.

Since *Pten/Trp53* double null mice died of enlarged prostate tumors by 5–6 months of age, we then assessed the sustained impact of Skp2 deficiency on prostate tumorigenesis of *Pten/Trp53* mutant mice. Remarkably, Skp2 deficiency significantly suppressed the growth of prostate tumors of *Pten^pc−/−^*; Trp53*^pc−/−^* mice (Supplementary Figure S1D). The average tumor mass of *Pten^pc−/−^*; Trp53*^pc−/−^*; Skp2**^−/−^ mice reduced 2.68-fold as compared to that of *Pten^pc−/−^*; Trp53*^pc−/−^* mice (Figure 1A, *P* < 0.001, N = 12 mice). Pathological analysis revealed that prostate tumors of *Pten^pc−/−^*; Trp53*^pc−/−^* mice developed poorly differentiated cancer (sarcomatoid) without discernible structures of prostate glands (Figure 1B). In contrast, prostate tumors of *Pten^pc−/−^*; Trp53*^pc−/−^*; Skp2**^−/−^ mice were much smaller with visible forms of glandular structures as HGPIN, and few cells displayed atypical nucleus. These results demonstrated that Skp2 inactivation restrains prostate cancer progression of *Pten/Trp53* mutant mice.

### Skp2 ablation reduces H3K4me3 levels through JARID1B to inhibit cell migration in MEFs

To delineate the impact of Skp2 in the regulation of H3K4me3, we first chose to examine the effect of Skp2 inactivation on the expression of H3K4me3 in Pten/Trp53 double null MEFs *in vitro*, and in *Pten^pc−/−^*; Trp53*^pc−/−^* mice *in vivo.* By following the same strategy reported previously [25, 26], we prepared Pten/Trp53 (*Pten^Δ/Δ^*; Trp53*^Δ/Δ^*) double null and Pten/Trp53/Skp2 triple null (*Pten^Δ/Δ^*; Trp53*^Δ/Δ^*; Skp2*^−/−^*) MEFs, and investigated the biological effects of Skp2 deficiency in Pten/Trp53 double null MEFs. In agreement with previous reports, a combined loss of *Pten* and *Trp53* genes in MEFs led to a significant increase of cell proliferation as compared to WT MEFs. Remarkably, the cell proliferation of Pten/Trp53/Skp2 triple null MEFs was significantly reduced as compared to Pten/Trp53 double null MEFs (Figure 1C). As Pten/Trp53 double null MEFs showed the soft agar transformation, we further assessed the suppressive effect of Skp2 inactivation on this malignant feature. Our results showed that Skp2 inactivation resulted in a significant reduction in colony size and numbers (Figure 1D, *P* < 0.01). In addition, Skp2 ablation resulted in a significant reduction of cell migration (the closure rate) (Figure 1E, *P* < 0.01, Supplementary Figure S1E).

We next evaluated H3K4me3 levels in Pten/Trp53 double null and Pten/Trp53/Skp2 triple null MEFs. Consistent with previous reports [7, 8], Skp2 deficiency resulted in an increased level of p27 protein in Pten/Trp53 double null MEFs (Data not shown). Importantly, Skp2 deficiency resulted in a significant reduction of H3K4me3 levels (3-fold), suggesting a pivotal role of Skp2 in the regulation of H3K4 trimethylation, at least in Pten and Trp53 double null background (Figure 1F). Meanwhile, Skp2 loss alone did not result in any reduction of H3K4me3 levels when compared to that in WT MEFs (Data not shown). Our results suggest that aberrant elevation of H3K4me3 levels by oncogenic insults may be a Skp2-dependent cascade. To investigate the mechanisms on the regulation of H3K4me3 by Skp2, we examined the effects of Skp2 ablation on the protein levels of JARID1B, a specific histone demethylase of H3K4me3/2 that is frequently overexpressed in PCa [17–20]. Western results revealed that JARID1B levels were aberrantly elevated upon the concomitant inactivation of both *Pten* and *Trp53* genes as compared to WT (Data not shown). Remarkably, Skp2 inactivation led to a striking elevation of JARID1B levels in Pten/Trp53 MEFs, and protein levels of JARID1B in Pten/Trp53/Skp2 triple null MEFs increased 2-fold as compared to that in Pten/Trp53 double null MEFs (Figure 1F, right panel), companying with a 3-fold decrease of H4K4me3 level. These data indeed provided biological evidence on a functional relationship between JARID1B and H3K4me3 in cells under defined oncogenic insults. Furthermore, our results revealed a novel function of Skp2 on the regulation of histone modification enzyme JARID1B to determine the levels of H3K4 trimethylation in cells. Taken together, these results demonstrated that Skp2 inactivation resulted in a reduction of H3K4me3, which may contribute to the inhibition of cell proliferation, transformation, and migration in MEFs.

### Skp2 deficiency decreases H3K4me3 levels in prostate tumors of *Pten/Trp53* mutant mice

**Figure 2:**
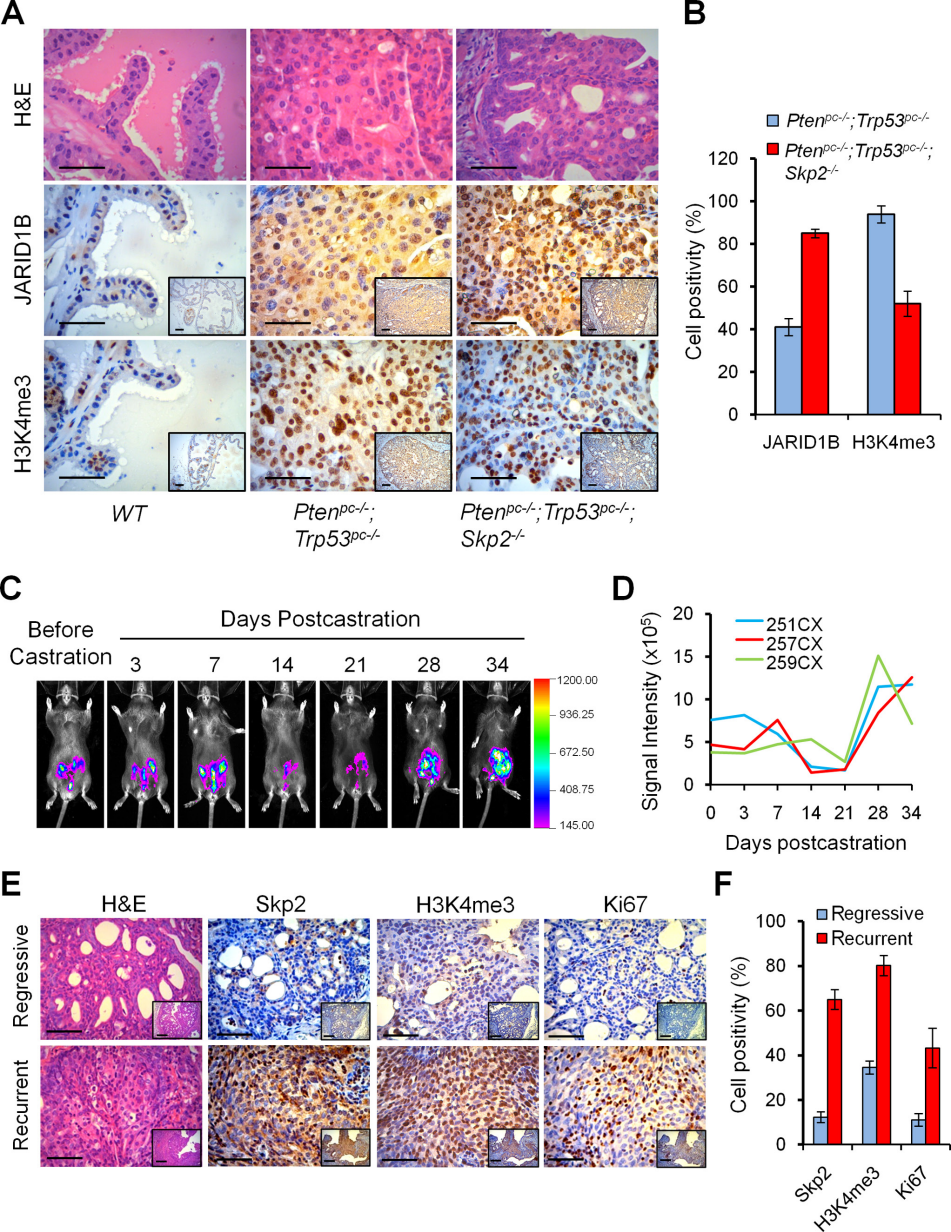
Skp2 inactivation decreases H3K4me3 to suppress prostate cancer progression *in vivo*, and H3K4me3 contributes to the recurrent growth of prostate tumors of *Pten/Trp53* mice **(A)** H&E and immunohistochemical (IHC) staining for H3K4me3 and JARID1B in prostate tumors of *Pten/Trp53* and *Pten/Trp53/Skp2* mutant mice. Scale bars represent 50 μm. **(B)** Quantification analysis of tumor cells positive for H3K4me3 and JARID1B. Error bars represent means ± SD from 3 mice for each group. **(C)** Modeling the recurrent growth of prostate cancer in *Luc/Pten/Trp53* null mice with bioluminescence imaging (BLI). The changes of BLI signals of representative *Luc/Pten/Trp53* mutant mouse were monitored prior to and post castration. Mouse castration was performed at 14 weeks of age. The regressive and recurrent tumor growth were monitored weekly and defined with the change of bioluminescence intensity in modeled mice (see Supplementary Figure S2). **(D)** Quantification of BLI signaling changes in prostate tumors of castrated *Luc/Pten/Trp53* mutant mice (*n* = 3). The signal intensity was measured for regions of interest around the lower abdomen. **(E)** IHC staining of Skp2, H3K4me3 and Ki67 in regressive and recurrent lesions of prostate tumor of *Luc/Pten/Trp53* mutant mice after castration. The regressive and recurrent tumor tissues of mice were collected at about 2 weeks or 3 weeks after castration, under the guidance of BLI. Scale bars represent 50 μm. **(F)** Quantification analysis of tumor cells positive for Skp2, H3K4me3 and Ki67. Error bars represent means ± SD from 3 mice for each group.

We then investigated whether the striking suppression of prostate tumorigenesis by Skp2 deficiency in *Pten^pc−/−^*; Trp53*^pc−/−^*; Skp2**^−/−^ mice was caused by an alteration of the H3K4me3/JARID1B coupling. Consistent with the findings *in vitro*, we discovered a reverse correlation between H3K4me3 and JARID1B levels in mouse prostate tumors *in vivo* (Figure 2A). Quantification analysis revealed that Skp2 inactivation indeed led to about 2-fold increase of JARID1B and consequently a marked 2-fold decrease of H3K4me3 levels in *Pten^pc−/−^*; Trp53*^pc−/−^*; Skp2**^−/−^ mice, as compared to *Pten^pc−/−^*; Trp53*^pc−/−^* mice (Figure 2B). Importantly, IHC staining results of consecutive sections revealed that the lesions with elevated accumulation of JARID1B in nucleus showed decreased levels of H3K4me3 (Figure 2A), indicating Skp2 may regulate JARID1B in PCa cells. These lines of evidence *in vitro* and *in vivo* support that Skp2 is an essential oncogenic factor for PCa progression by driving the dysregulation of H3K4 trimethylation through JARID1B.

### H3K4me3 correlates with the recurrent growth of prostate tumors in *Pten/Trp53* mice

Aberrations of histone methylation and acetylation are associated with clinical outcome in several cancers [27, 28]. Elevated expression of H3K4me3 is frequently found in CRPC as compared to benign tumor specimens [17], suggesting the dysregulation of H3K4 trimethylation may contribute to castration resistance. Therefore, we wished to obtain new insights into the dysregulation of H3K4me3 on CRPC using mouse models. To do so, we generated luciferase-labeled *Pten/Trp53* conditional double null (*Luc^pc+^*;*Pten^pc−/−^*;Trp53*^pc−/−^* referred to *Luc/Pten/Trp53*) mutant mice, in which the expression of firefly *luciferase* gene is turned on concomitantly with the inactivation of *Pten* and *Trp53* genes in prostatic epithelium driven by *Probasin-Cre4* after puberty [25, 29]. As shown in the biopsy and the bioluminescence imaging (BLI), the luciferase positivity was confined to prostate tumors of mutant mice (Supplementary Figure S2). Therefore, we used *Luc/Pten/Trp53* mutant mice to recapitulate features of human CRPC by performing castration at 14 weeks of age when most *Pten^pc−/−^*;Trp53*^pc−/−^* mice developed invasive cancer [25, 29, 30]. As revealed by BLI, prostate tumors of *Luc/Pten/Trp53* mutant mice displayed a striking tumor regression at 2–3 weeks post castration and then regained tumor growth (Figure 2C and 2D). All mutant mice died of (or were sacrificed due to) enlarged prostate tumors from the recurrent growth by 5–6 months of age. In order to understand the molecular profiles on the recurrent growth of PCa, we collected the prostate tissues for histological analysis after regression or when recurrence as demonstrated by luciferase signal intensity at pertinent time points post-castration. Remarkably, IHC staining revealed that Skp2 accumulation was significantly higher in recurrent tumors as compared to regressive tumors in mice (Figure 2E and 2F), suggesting a critical role of Skp2 in CRPC. We then examined whether elevated Skp2 was associated with the dysregulations of H3K4me3 proteins in CRPC. Importantly, H3K4me3 was indeed dramatically upregulated in recurrent tumors, when compared to that in regressive tumors (Figure 2E and 2F). This expression pattern of H3K4me3 in mouse models is in agreement with the previous report on H3K4me3 elevation in human hormone-refractory PCa [17]. These results provide *in vivo* evidence to support that the dysregulation of H3K4me3 by SKP2 contributes to CRPC growth.

### SKP2 determines H3K4me3 by attenuating K63-linked ubiquitination of JARID1B

**Figure 3:**
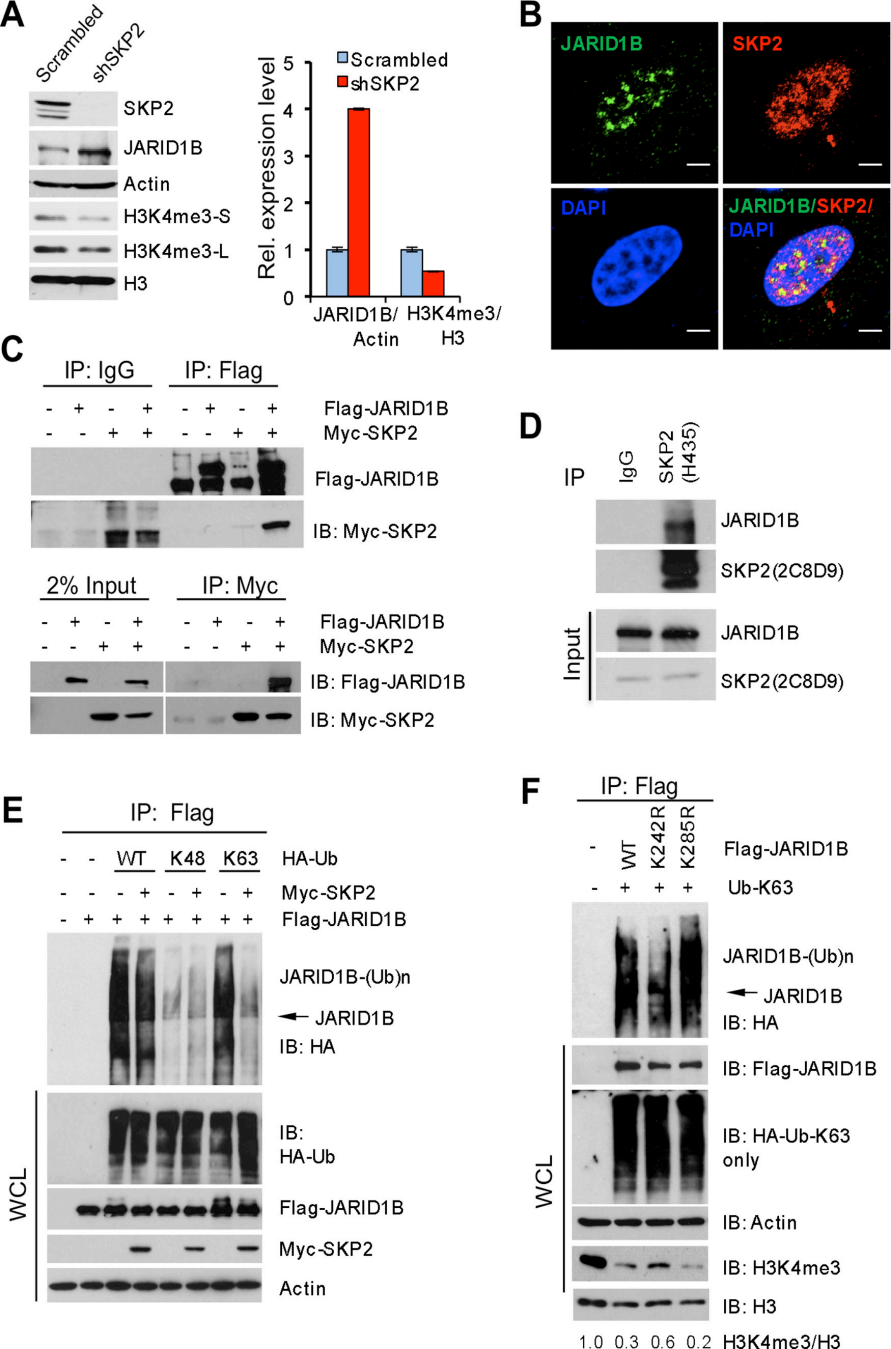
SKP2 suppresses K63-linked ubiquitination of JARID1B to elevate H3K4me3 in human prostate cancer cells **(A)** Western blot analysis shows JARID1B elevation and H3K4me3 reduction in PC3 cells upon SKP2 knockdown by shRNA. S - short exposure, L - long exposure. Right panel: quantification analysis on the abundance of H3K4me3 and JARID1B proteins from the left panel. Error bars represent means ± SD. **(B)** Immunofluorescence images show the co-localization of endogenous SKP2 and JARID1B proteins in PC3 cells. Scale bars represent 10 μm. **(C)** Co-immunoprecipitation analysis shows a physical interaction between JARID1B and SKP2 proteins in HEK293T cells. **(D)** Co-immunoprecipitation analysis shows a physical interaction between endogenous JARID1B and SKP2 proteins in PC3 cells. **(E)** *In vivo* ubiquitination assay shows that a reduction of K63-linked ubiquitination of JARID1B upon addition of SKP2 in HEK293T cells. Cells were transfected with Flag-JARID1B, Myc-SKP2, along with various HA-ubiquitin (HA-Ub) constructs. K48 and K63 indicate HA-Ub-K48-only and HA-Ub-K63-only, respectively. WCL indicates whole cell lysates. **(F)** JARID1B mutation affects H3K4me3 levels by altering the ubiquitination. In vivo ubiquitination assay was performed in HEK293T cells transfected with HA-Ub-K63-only, along with various JARID1B constructs (Flag-JARID1B, Flag-JARID1B-K242R and Flag-JARID1B-K285R) (see Supplementary Figure S4). Bottom panel: Western blot analysis shows the effects of JARID1B WT and JARID1B mutants on the changes of endogenous protein levels of H3K4me3 through the K63-linked ubiquitination in cells. WCL indicates the whole cell lysates.

We then investigated mechanisms underlying the dysregulation of H3K4me3/JARID1B by SKP2 using human PCa cells. At first, we assessed the levels of SKP2, JARID1B and H3K4me3 proteins in several human PCa cell lines. The levels of SKP2, JARID1B and H3K4me3 proteins are highly expressed in LNCaP, C4-2B, CWR22RV1 and PC3 cells (Supplementary Figure S3A). Therefore, to understand the effect of SKP2 ablation on JARID1B, we chose PC3 cells to knock down SKP2 using a lentiviral-based small hairpin RNA (shRNA) technology because PC3 cells show a high level of endogenous SKP2. As confirmed by Western blot, SKP2 knockdown completely abolished the protein expression of SKP2 in PC3-shSKP2 cells as compared to that in control cells (Figure 3A). Remarkably, H3K4me3 levels in PC3-shSKP2 cells were decreased 50% as compared to that in control cells (Figure 3A, right panel). Most importantly, SKP2 ablation resulted in a striking increase of JARID1B compared to the control. Quantification analysis revealed that JARID1B levels in PC3-shSKP2 cells were 4-fold higher than that in control cells (Figure 3A, right panel). These results from human PCa cells were in agreement with our data from MEFs (Figure 1F).

We reasoned that SKP2 might directly regulate the histone modification enzyme JARID1B at either the transcription or the post-transcription level. At first, we examined the changes of JARID1B mRNA upon SKP2 knockdown. Our qRT-PCR results showed that mRNA levels of JARID1B in PC3-shSKP2 cells were comparable to that in control cells (Supplementary Figure S3B), indicating that the elevation of JARID1B protein in PCa cells upon SKP2 knockdown may be caused at the post-transcription level. We then assessed the effects of SKP2 knockdown on the stability of JARID1B protein. Surprisingly, we found that SKP2 knockdown in PC3 cells indeed resulted in a prolonged half-life of JARID1B protein. The half-life of JARID1B protein in PC3-shSKP2 cells was about 12 hr as compared to 6 hr in control cells (Supplementary Figure S3C).

We wished to understand whether SKP2 protein interacts with JARID1B to regulate H3K4me3 levels in PCa cells. Immunofluorescence (IF) results revealed a co-localization of endogenous SKP2 and JARID1B proteins in nucleus of PC3 cells (Figure 3B), implicating their physical interaction. Similarly, the co-localization of JARID1B and SKP2 was found when Flag-JARID1B and Myc-SKP2 were ectopically expressed in HEK293T cells (Supplementary Figure S3D, bottom panel). Interestingly, a reverse correlation between JARID1B and SKP2 protein levels was observed endogenously in PC3 cells as well as exogenously in HEK293T cells (Supplementary Figure S3D), suggesting that SKP2 regulates JARID1B levels in cells. To confirm this interaction, HEK293T cells were co-transfected with Flag-JARID1B and Myc-SKP2 plasmids, and a co-immunoprecipitation was performed using anti-Flag or anti-C-Myc. As shown, SKP2 protein was indeed detected in the immunoprecipitates with anti-Flag for JARID1B in cells cotransfected with both JARID1B and SKP2 plasmids, whereas no coimmunoprecipitation between JARID1B and SKP2 proteins was found in cells co-transfected with empty vectors or Myc-SKP2 plasmid alone (Figure 3C, top panel). In addition, JARID1B protein was detected in the immunoprecipitates when a reciprocal co-immunoprecipitation was performed using anti-Myc antibody for SKP2 (Figure 3C, bottom panel). Most importantly, the physical interaction between these two proteins was demonstrated by a co-immunoprecipitation of endogenous SKP2 and JARID1B in PC3 cells (Figure 3D). These data provided solid biochemical evidence on a physical interaction between JARID1B and SKP2 proteins in cells, supporting that the function role of SKP2 on antagonizing JARID1B protein for H3K4me3 elevation in cancer cells.

Since SKP2 is an E3 ubiquitin ligase for the degradation of several proteins through ubiquitination [2, 3, 31], we performed *in vivo* ubiquitination assay to explore whether SKP2 may also target JARID1B for the ubiquitin-mediated degradation. This would help provide a mechanistic explanation on the potential antagonizing role of SKP2 on the stability of JARID1B protein. HEK293T cells were cotransfected with SKP2, JARID1B and HA-tagged ubiquitin or ubiquitin mutants including K48-only and K63-only ubiquitin plasmids. Surprisingly, our results revealed that the polyubiquitination of JARID1B protein was conjugated through ubiquitin at lysine 63 instead of lysine 48, the common form of polyubiquitination for proteasome-dependent protein degradation (Figure 3E). Surprisingly, we observed that addition of SKP2 failed to promote, but reduced, JARID1B ubiquitination contributed by WT ubiquitin and ubiquitin K63-only mutant. Lysine 48(K48)-linked ubiquitination is generally contributing to protein degradation through ubiquitin-mediated proteasome pathway, while lysine 63(K63)-linked ubiquitination is responsible for various protein functions including kinase activity and protein trafficking [32]. Our results suggest that SKP2 may have a novel regulatory role on K63-linked polyubiquitination of JARID1B, in addition to its E3 ubiquitin ligase activity.

To define the biological relevance of K63-linked JARID1B ubiquitination in PCa cells, we decided to generate JARID1B mutants to see the impact on H3K4me3 levels, the enzymatic product of JARID1B. UbPred program predicted that lysine residues 242 and 285 are potential ubiquitination sites of JARID1B (Supplementary Figure S4) [33]. We generated JARID1B mutants by site-directed mutagenesis and then performed *in vivo* K63-linked ubiquitination assay. Our results demonstrated that JARID1B mutant at lysine 242 (K242R) but not at lysine 285 (K285R) resulted in a dramatic decrease of K63-linked ubiquitination of JARID1B, when compared to that of wild type JARID1B (WT) (Figure 3F), suggesting lysine 242 in JARID1B may be one of the major conjugation sites for polyubiquitination through lysine 63 of ubiquitin. Furthermore, we examined the effects of K63-linked polyubiquitination of JARID1B on the levels of H3K4me3. Our results showed that overexpression of JARID1B WT or K285R mutant, resulted in a dramatic reduction of H3K4me3 level to 30% and 20%, respectively, as compared to the control, the endogenous level of H3K4me3 in HEK293T cells. In contrast, K242R mutant of JARID1B only reduced H3K4me3 level to 60% compared to the control, resulting in 2 or 3-fold elevation of H3K4me3 levels than that of JARID1B-WT and JARID1B-K285R (Figure 3F). This result is consistent with the inhibition of K63-linked ubiquitination of JARID1B by K242R mutation in the presence of Ub-K63-only proteins. These lines of biochemical evidence indicate that K63-linked ubiquitination of JARID1B is required for its enzymatic activity to demethylate H3K4me3.

### SKP2 downregulates TRAF6-mediated ubiquitination of JARID1B

**Figure 4:**
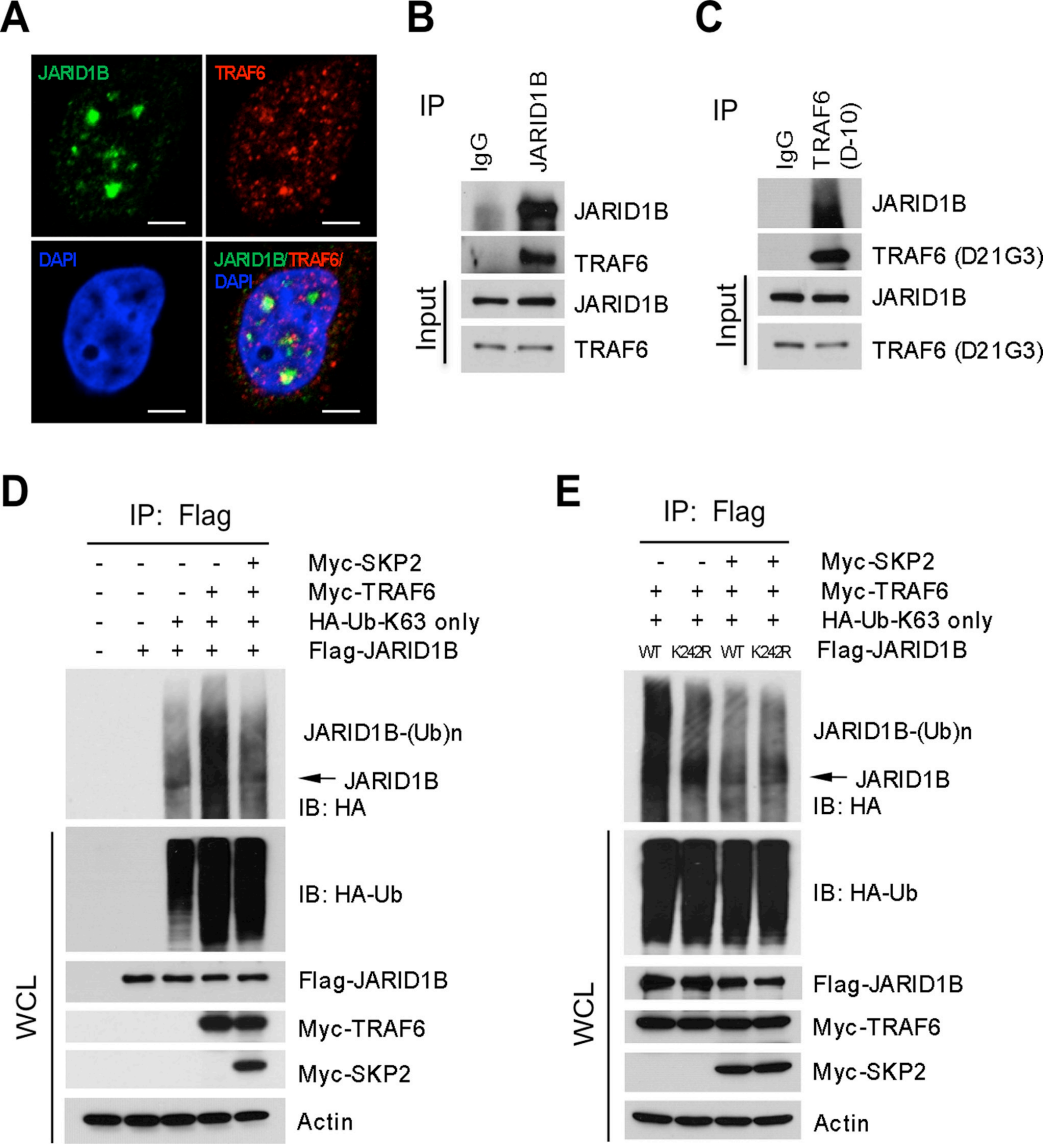
SKP2 regulates the ubiquitination of JARID1B through TRAF6 **(A)** Immunofluorescence images show a co-localization of endogenous JARID1B and TRAF6 in PC3 cells. Scale bars represent 10 μm. **(B)** and **(C)** Co-immunoprecipitation analysis shows that endogenous TRAF6 physically interacts with JARID1B in PC3 cells, as shown by reciprocal co-immunoprecipitation between the two proteins (Also see Supplementary Figure S5). **(D)** *In vivo* ubiquitination assay shows that TRAF6 increases K63-linked ubiquitination of JARID1B and SKP2 inhibits TRAF6-mediated JARID1B ubiquitination. Cells were transfected with Flag-JARID1B, HA-Ub-K63-only, Myc-TRAF6 and Myc-SKP2 constructs as indicated. WCL indicates the whole cell lysates. **(E)** TRAF6 mediates JARID1B ubiquitination through lysine residue 242. HEK293T cells were transfected with Flag-JARID1B WT or Flag-JARID1B-K242R, HA-Ub-K63-only, Myc-TRAF6 and Myc-SKP2 plasmids as indicated. *In vivo* ubiquitination assay was performed in a standard procedure. WCL indicates the whole cell lysates.

Since JARID1B ubiquitination is not directly catalyzed by SKP2, we deduced that SKP2 might regulate other E3 ubiquitin ligases responsible for the K63-linked ubiquitination of JARID1B to indirectly affect H3K4me3 levels. We reasoned that TRAF6 is a candidate E3 ubiquitin ligase for K63-linked ubiquitination of JARID1B, as it is mainly involved in the synthesis of polyubiquitin chains linked through ubiquitin lysine 63. Literature reports that TRAF6 is required to promote TGF-β induced apoptosis of PCa cells through activation of TGF-β-Associate Kinase 1(TAK1) in TGF-β signaling pathway [34, 35]. *In silico* amino acid sequence analysis revealed that JARID1B contains three potential consensus TRAF6 binding motifs (PxExxAr/Ac), which are adjacent to the ubiquitination site of JARID1B at lysine 242 (Supplementary Figure S5A). IF assay showed a co-localization of TRAF6 and JARID1B proteins, suggesting the existence of their physical interaction (Figure 4A). We next performed reciprocal co-immunoprecipitation assays with anti-TRAF6 or anti-JARID1B antibody using lystates of PC3 cells that contain both endogenous JARID1B and TRAF6. Our data showed that these two proteins physically interact, suggesting that TRAF6 may be an E3 ubiquitin ligase for the K63- linked ubiquitination of JARID1B (Figure 4B and 4C). To confirm this possibility, we performed *in vivo* ubiquitination assay with HEK293T cells that were cotransfected with TRAF6, JARID1B or JARID1B mutants and ubiquitin-K63-only mutant plasmids, in the absence or the presence of SKP2. As expected, TRAF6 dramatically increased the K63-linked polyubqiuitination of JARID1B, and addition of SKP2 resulted in a striking reduction of the polyubiquitination (Figure 4D). Furthermore, JARID1B K242R mutation abrogated TRAF6-mediated ubiquitination of JARID1B (Figure 4E).

We investigated the relationship between SKP2 and TRAF6 to elucidate the role of SKP2 on TRAF6-mediated JARID1B ubiquitination in PCa cells. Western blot analysis revealed that TRAF6 protein levels were markedly elevated in PC3 cells upon SKP2 knockdown as compared to the control (Supplementary Figure S5B). In addition, IHC results also showed that TRAF6 levels were strikingly increased in prostate tumors of *Pten^pc−/−^*; Trp53*^pc−/−^*; Skp2**^−/−^ mice, as compared to that of *Pten^pc−/−^*; Trp53*^pc−/−^* mice (Supplementary Figure S5C). These data support that SKP2 maintains a low level of TRAF6 protein both *in vitro* and *in vivo*. In summary, our results disclose a novel mechanism by which SKP2 indirectly inhibits the K63-linked ubiquitination of JARID1B via E3 ubiquitin ligase TRAF6.

### SKP2 deficiency promotes ubiquitinated JARID1B in nucleolus of PCa cells for senescence *in vitro* and *in vivo*

**Figure 5:**
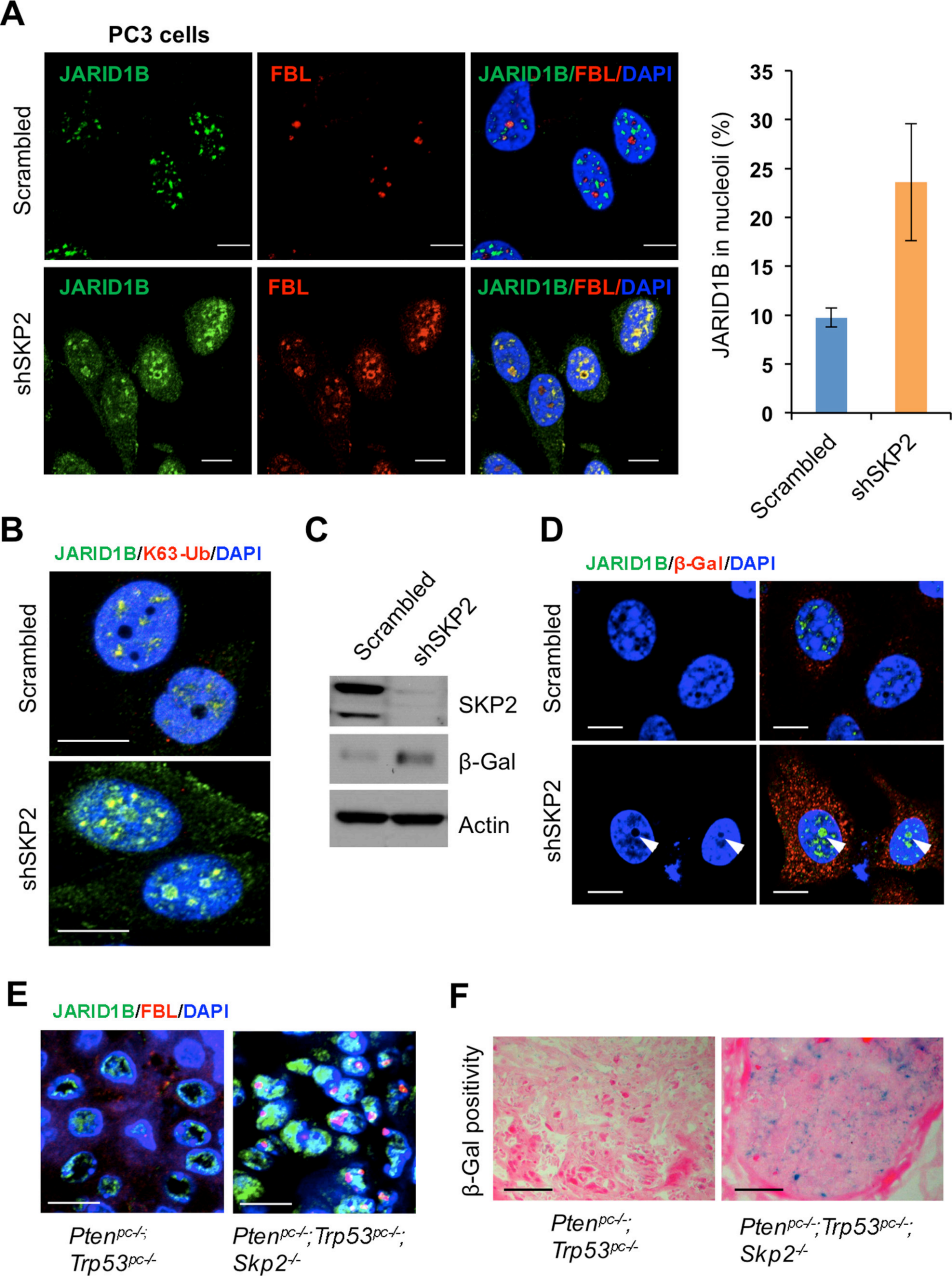
SKP2 inactivation induces an accumulation of ubiquitinated JARID1B in nucleolus of cells *in vitro* and *in vivo* for cellular senescence **(A)** Immunofluorescence (IF) images show a co-localization of endogenous JARID1B and Fibrillarin (FBL) in nucleoli of PC3 cells upon SKP2 knockdown (Also see Supplementary Figure S6A). FBL indicates a nucleolar marker. Right panel: quantification of PC3 cells showing an increase of JARID1B localization in nucleolus. Error bars represent means ± SD. **(B)** IF images show a co-localization of endogenous JARID1B and K63-Ub in nucleoli of PC3 cells upon SKP2 knockdown. **(C)** Western blotting assay shows an increase of β-galactosidase (β-Gal) in PC3 cells upon SKP2 knockdown. **(D)** IF images show JARID1B in nucleolus as indicated by arrows and β-Gal in cytoplasm in senescent cells upon SKP2 knockdown. **(E)** The co-localization of endogenous JARID1B and Fibrillarin (FBL) in nucleoli of prostate tissues in *Pten^pc−/−^*;Trp53*^pc−/−^*;Skp2*^−/−^* mutant mice (Also see Supplementary Figure S6B). Scale bars represent 10 μm for panel A, B, D and E. **(F)** The positive staining of β-galactosidase in prostate tissues of *Pten^pc−/−^*;Trp53*^pc−/−^*;Skp2*^−/−^* mice but not in that of *Pten^pc−/−^*;Trp53*^pc−/−^* mice. Scale bars represent 50 μm.

Our finding on the K63-linked ubiquitination of JARID1B mediated by SKP2 promoted us to hypothesize that SKP2 may also contribute to histone modifications by regulating protein trafficking of JARID1B in PCa cells. To test this hypothesis, we performed IF analyses to inquire the subcellular localization of JARID1B in PC3-shSKP2 cells compared to that in PC3-scrambled cells. Unexpectedly, we observed an altered distribution of JARID1B protein in nucleus of PC3 cells upon SKP2 knockdown, indicating that the trafficking of JARID1B protein was restrained inside nucleus, very little to cytoplasm (Figure 5A and Supplementary Figure S6A and S8A). Fibrillarin (FBL), a major component of ribonuceloproteins in nucleolus, is recently reported to associate with histone modifications [36]. Therefore, we decided to examine whether JARID1B protein shuttled to nucleoli of PC3 cells upon SKP2 knockdown. As shown, we found that JARID1B is predominantly localized in the nucleoli of PC3-shSKP2 cells upon SKP2 knockdown as evidenced by its co-localization with FBL, whereas JARID1B is largely localized in the nuceloplasm of PC3-scrambled cells (Figure 5A and Supplementary Figure S6A, arrows in white color). Moreover, we sought for the nucleolar localization signal (NoLS) in JARID1B protein, and found four potential NoLS sequences predicted by NOD program (Supplementary Figure S7). For a validation of NOD program, human p14ARF-a known nucleolar protein was tested as a control, and genuine NoLS signal sequences were identified (Supplementary Figure S7) [37]. To confirm that JARID1B protein in nucleolus still remains in the K63-linked ubiquitination, we performed IF staining with K63-Ub antibody. Our results showed that K63-Ub was co-localized with JARID1B in the nucleolus of PC3-shSKP2 cells, but not in that of PC3-scrambled cells (Figure 5B and Supplementary Figure S8B). These results indicate that the K63-linked ubiquitination of JARID1B may be required for JARID1B protein to shuttle to nucleoli for biological functions upon SKP2 knockdown.

SKP2 deficiency induces cellular senescence in contexts of oncogenic insults [8], and recently JARID1B is associated with cellular senescence in colorectal cancer and retinoblastoma cells [38, 39]. We reasoned that K63-linked ubiquitination of JARID1B accumulated in nucleolus may contribute to SKP2-deficient induced cellular senescence. To test this hypothesis, we assessed the protein level of β-galactosidase, a senescence marker, in PC3-shSKP2 cells. As predicted, a striking increase of β-galactosidase was found in PC3-shSKP2 cells, as compared to that in PC3-scrambled cells (Figure 5C), in agreement with literature that SKP2 deficiency induces cellular senescence. Importantly, IF results revealed that a remarkable accumulation of JARID1B protein was found in nucleolus of senescent PC3-shSKP2 cells but not in non-senescent PC3-srambled cells (Figure 5D). These results indicate that nucleolar JARID1B may contribute to the induction of cellular senescence in human PCa cells. Our findings *in vitro* on the SKP2 deficiency-promoted shuttling of JARID1B protein encouraged us to validate the phenomenon in prostate tumors of mice. Our results showed that JARID1B and FBL proteins are co-localized in a majority of epithelial cells in prostate tumors of *Pten^pc−/−^*; Trp53*^pc−/−^*; Skp2**^−/−^ mice, but not in that of *Pten^pc−/−^*; Trp53*^pc−/−^* mice (Figure 5E and Supplementary Figure S6B). Similarly, the co-localization of JARID1B and K63-Ub proteins was also found in prostate epithelial cells of *Pten^pc−/−^*; Trp53*^pc−/−^*; Skp2**^−/−^ mice (Supplementary Figure S8C). The cellular senescence was detected in prostate tumors of *Pten^pc−/−^*; Trp53*^pc−/−^*; Skp2**^−/−^ mice, but not in that of *Pten^pc−/−^*; Trp53*^pc−/−^* mice (Figure 5F).

Taken together, we demonstrated that SKP2 restricts JARID1B trafficking to nucleoli of PCa cells through attenuating its K63-linked ubiquitination. The K63-linked ubiquitination procedure is likely required for the nucleolar shuttling to maintain the function of JARID1B for demethylation of H3K4me3 and for the regulation of cellular senescence in PCa cells. SKP2 deficiency suppresses PCa by altering JARID1B ubiquitination, shuttling, and thus contributing to epigenetic modifications of H3K4me3 and cellular homeostasis.

### H3K4me3 levels are positively correlated with SKP2 expression in human prostate cancer

**Figure 6:**
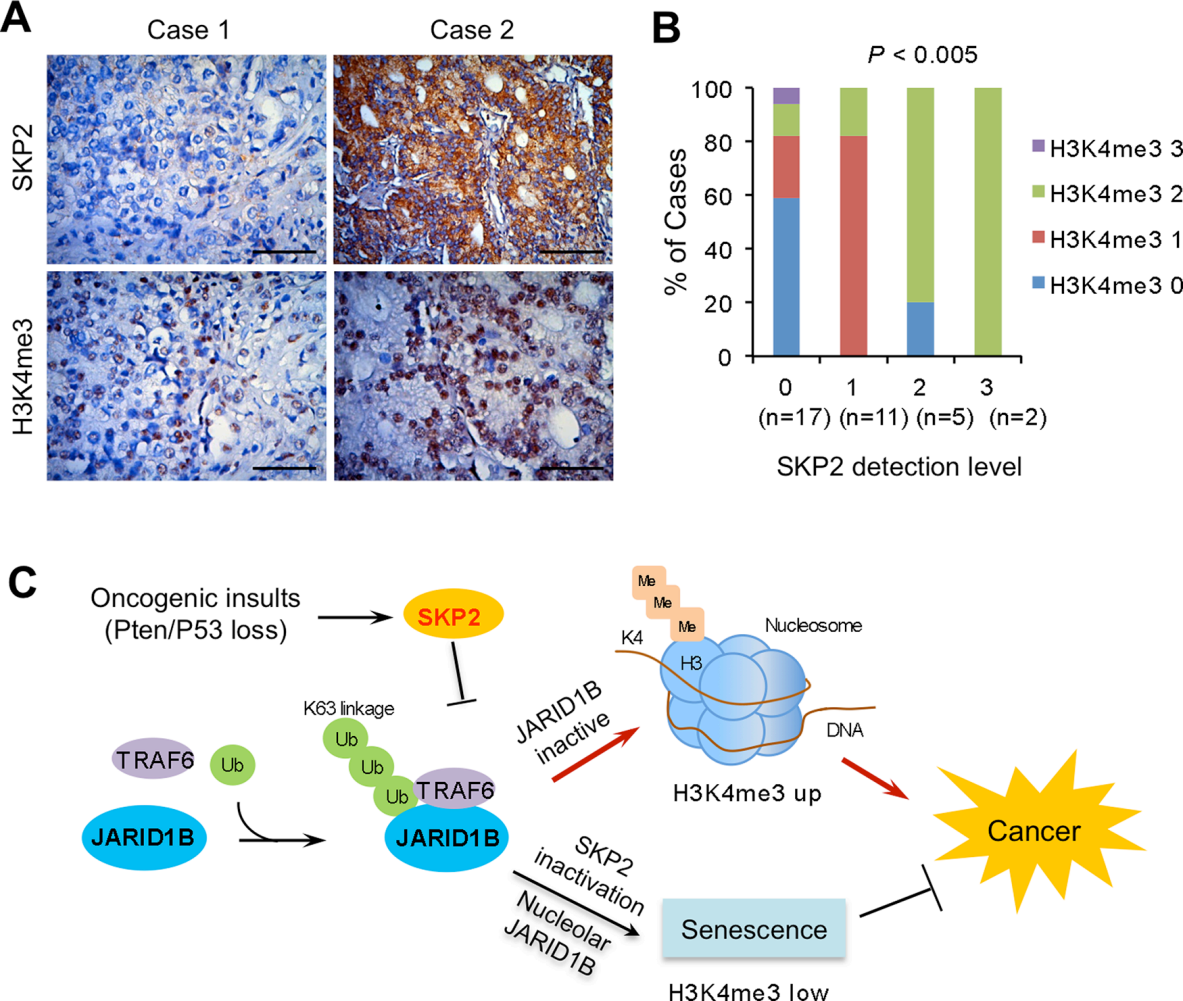
SKP2 positively correlates with H3K4me3 in human prostate cancer specimens **(A)** Immunohistochemical staining on SKP2 and H3K4me3 in human prostate array tissues. Scale bars represent 50 μm. **(B)** Statistical analysis of the prostate tissue microarray stained with SKP2 and H3K4me3 antibodies. The percentages of different H3K4me3 levels were calculated for each level of SKP2 protein in 35 cases of human PCa specimens. SKP2 and H3K4me3 levels were graded as 0, 1, 2 and 3 by intensity scores. The H3K4me3 grades are color-coded. Numbers in parenthesis represent sample sizes. The statistical significance was determined by Chi-Square test (Table S2). **(C)** A working model for the role of SKP2 on epigenetic regulation of JARID1B and H3K4me3 in PCa. SKP2 suppresses the activity of JARID1B by reducing its K63-linked ubiquitination through TRAF6 under oncogenic stimulation, leading to an elevated H3K4me3 thus contributes to PCa. SKP2 deficiency increases JARID1B transportation to nucleolus of cells through an increase of its ubiquitination, resulting in an induction of cellular senescence to suppress PCa tumorigenesis.

To understand the correlation between SKP2 and H3K4me3 as well as the relevance in human PCa, we performed IHC staining of SKP2 and H3K4me3 in human prostate tissue microarrays (TMA) consisting of cancer and normal cases. The elevation of SKP2 protein was found in both cytoplasm and nucleus, while H3K4me3 was primarily accumulated in the nucleus of cancer cells (Figure 6A). In addition, the correlation between SKP2 and H3K4me3 levels was assessed by Pearson correlation test, with grades taken as continuous variables, which was further validated by Chi-square tests. Results from both statistical analyses indicated that SKP2 expression was positively correlated with H3K4me3 levels (Pearson correlation coefficient = 0.4693, *P* = 0.0044; χ2 test, *P* = 0.0006) in PCa (Figure 6B and Supplementary Table S2), when graded in intensity staining scores, respectively. These data suggest the deregulation of SKP2 and H3K4me3 plays essential roles in human PCa.

## DISCUSSION

Aberrant elevation of histone post-translational modifications is recognized to make important contributions to the initiation and progression of human cancers by altering the expression profile of multiple genes [18, 27]. The dysregulation of H3K4me3, one of the most important histone methylations, has been found to associate with the development of cancers including PCa [18]. Given that H3K4me3 levels are mainly determined by JARID1B, it is crucial to understand molecular mechanisms on the regulation of JARID1B and the relevance in PCa. Yet, due to the limits of current technology and lack of authentic mouse models, dysregulation of H3K4me3 and JARID1B *in vivo* upon oncogenic insults remains poorly understood [19]. Our studies with *in vitro* and *in vivo* evidence added valuable knowledge to decode this phenomenon in 3 ways: 1) Skp2 ablation results in a reduction of H3K4 trimethylation and a suppression of cancer progression; 2) JARID1B activity requires its K63-linked ubiquitination, which is suppressed by elevated SKP2 through regulating E3 ubiquitin ligase TRAF6 in PCa cells; and 3) K63-linked ubiquitination of JARID1B may be required for protein trafficking into nucleolus to control cellular homeostasis through senescence mechanism. Our results provide new and valuable mechanistic insights into understanding the regulation of H3K4me3 and JARID1B and further effects of oncogenic protein SKP2 on PCa.

Both SKP2 elevation and aberrant histone modifications are frequently detected in CRPC and recognized as important markers for cancers [27, 40, 41]. SKP2 is required to maintain the normal cell cycle and its inactivation results in cell cycle arrest [2]. Importantly, upon oncogenic insults, SKP2 inactivation drives cell cycle arrest to irreversible senescence (also termed as geroconversion) [8, 42]. However, the connection between SKP2 elevation and histone modifications remains unclear [18]. In this study, we applied genetic approaches to dissect the molecular contributions of SKP2 to epigenetic alterations in prostate tumorigenesis. This notion is supported with *in vitro* evidence that Skp2 deficiency reduces cell proliferation, transformation and migration of *Pten/Trp53* double null MEFs (Figure 1), and with *in vivo* evidence that SKP2 deficiency constrains the initiation and growth of prostate tumors of *Pten^pc−/−^*; Trp53*^pc−/−^* mice (Figure 1 and Supplementary Figure S1). Importantly, we discovered that Skp2 elevation was highly correlated with the increased levels of H3K4me3 in primary (Data not shown) and recurrent PCa tissues in mice upon oncogenic stimulation (Pten/Trp53 loss) (Figure 2E), whereas Skp2 ablation dramatically reduced H3K4me3 (Figure 1F and 2A). These results indicate the abnormal elevation of H3K4me3 depends on Skp2, at least in Pten/Trp53 null background, which implicating that SKP2 plays an essential role in dysregulation of H3K4me3 for the development and progression of cancers. Our findings are in harmony with previous reports that SKP2 has a critical pro-oncogenic function for the development and progression of cancers including PCa, through affiliating oncogenic signaling pathways such as *Rho*A, c-Myc and Ras [6, 8, 10]. It will be worthy to evaluate if the dysregulation of H3K4 trimethylation depends on Skp2 in other mouse models of cancers, and furthermore the relevance in mTOR signaling pathway [43].

Methylation, acetylation and phosphorylation of histones contribute to cancers and diseases in humans by synchronizing the epigenetic modification cascades to mediate the functions of oncogenes and tumor suppressor genes in cells [44–47]. On the other hand, aberrant histone modifications are substantially associated with aberrant expression and mutations of epigenetic modification regulators in human cancers [17, 18]. We revealed a novel molecular mechanism in which SKP2 determines the levels of H3K4me3 by mediating K63-linked ubiquitination of JARID1B, a key demethylase of H3K4me3, through regulating E3 ubiquitin ligase TRAF6. The K63-linked ubiquitinated form of JARID1B interacts with SKP2 in the nucleoplasm of cells when SKP2 is present or elevated, but sharply increases and shuttles to the nucleolus of PCa cells upon SKP2 ablation (Figure 5 and Supplementary Figure S6 and S8). Most recently, Tessarz et al. showed that nucleolus is involved in histone modifications [36], so it is likely that JARID1B/H3K4me3 coupling requires Fibrillarin in nucleolus under this setting. Paradoxically, the protein level of JARID1B increases upon SKP2 loss in both MEFs and human PCa cells, suggesting the JARID1B protein stability may be mediated by SKP2 through ubiquitination mechanisms independent of lysine 48. For example, literature reports that sumoylated JARID1B/KDM5B on lysine 242 can be marked by E3 SUMO ligase RNF4 for proteasome mediated degradation [48]. In this study, we observed that the lysine 242 of JARID1B is ubiquitinated through ubiquitin K63-linkage, suggesting that JARID1B protein may be differentially regulated by synchronization of the ubiquitination and sumoylation machineries in cancers. It is likely that an increase of JARID1B ubiquitination in the absence of SKP2 will competitively decrease the free lysine 242 for the sumoylation-mediated degradation by RNF4.

JARID1B/KDM5B/PLU1, the H3K4me3/2 demethylase, is frequently elevated in advanced PCa [19, 20, 49] and associated with multidrug resistance [50], underscoring its potential oncogenic roles in cancers. The mechanisms leading to JARID1B elevation and possible driving pathways to cancers, in addition to its demethylase function, are still paucity of understanding. In our study, we unveiled *in vitro* and *in vivo* that JARID1B was regulated by SKP2 at a post-translational fashion using several biological systems including human PCa cells, MEFs, and mouse models. Our results showed that SKP2 had significant effects on JARID1B activity and H3K4me3 levels. SKP2 knockdown or deficiency results in an increase of JARID1B protein level, which partially in line with the literature that SKP2 is an important component of SCF complex functioning for protein degradation [7, 51]. Importantly, we found that SKP2 elevation is positively correlated with H3K4me3 in PCa specimens (Figure 6A and 6B). Taken together, our study revealed a novel function of SKP2 in mediating JARID1B for histone methylations (Figure 6C), and further investigation is needed to decipher the molecular interactions among SKP2, TRAF6 and JARID1B proteins.

In short, our studies revealed a novel mechanism in which JARID1B is regulated by SKP2 through K63-linked ubiquitination, which leading to aberrant alterations of H3K4me3 in turmorigenesis and CRPC. Furthermore, our results, together with previous reports, support that Skp2 deficiency restrains cell proliferation and tumor progression by triggering cellular senescence [8], chromatin remodeling and histone modifications [39]. Our findings on SKP2 and JARID1B suggest that a combinatory targeting on SKP2 and JARID1B may be more potent chemotherapy for CRPC treatment.

## MATERIALS AND METHODS

### Mutant mice, genotyping and tumor analysis

*Pten^loxP/loxP^*; *Trp53^loxP/loxP^*; *Skp2*^−/−^ and *Probasin-Cre4* mutant mice were generated and maintained as previously described [8, 25]. Briefly, in order to obtain desired genotypes of compound mutant mice, female mice carrying alleles of *LoxPten*, *LoxTrp53*, and *Skp2^+/−^* were crossed with male mice carrying alleles of *LoxPten*, *LoxTrp53*, *Skp2^+/−^* and *Probasin-Cre4* to produce conditional double knockout mutants *Pten^loxP/loxP^*; Trp53^loxP/loxP^*; *Probasin-Cre4* (referred to *Pten^pc−/−^*; Trp53*^pc−/−^*) and conditional triple knockout mutants *Pten^loxP/loxP^;Trp53^loxP/loxP^*; *Skp2*^−/−^*; *Probasin-Cre* (referred to *Pten^pc−/−^*; Trp53*^pc−/−^*; *Skp2^−/−^* or *Pten/Trp53/Skp2*). To generate the prostate-specific luciferase *Pten/Trp53* double null mutant mice, luciferase transgenic female mice (*Luc^ROSA26 LSL^*) were crossed with *Pten^loxP/+^*;*Trp53^loxP/loxP^*; *Probasin-Cre4* male mice. Their F2 offspring was further used to generate *Luc^LSL^*; *Pten^loxP/loxP^*; *Trp53^loxP/loxP^*; *Probasin-Cre4* (*referred to Luc^pc+^*; *Pten^pc−/−^*; Trp53*^pc−/−^* or *Luc/Pten/Trp53*) compound mutants. All experimental animals were kept in a mixed genetic background of C57BL/6J X 129sv X DBA2, and animal experiments were conducted in accordance with an IACUC-approved protocol at Meharry Medical College.

All genotypes were verified by performing polymerase chain reaction (PCR) with DNA extracted from mouse tails according to protocols previously described [8, 25]. Briefly, mouse tail tissues at 0.5cm in length were digested in a volume of 300 μl lysis buffer containing Proteinase K (350 μg/ml) over night at 55°C, and DNA was precipitated with 2 volumes of ethanol and then suspended in 500 μl of ddH_2_O for use. Primers used for genotyping PCR were previously described and listed in Supplementary Table S1. PCR programs were run as 95°C for 4 min, then 95°C 30s, 57°C 1 min, 72°C 1 min for 34 cycles, with a final elongation at 72°C for 7 min in a BioRad thermal cycler.

Mice in indicated genotypes were sacrificed at 3 or 6 months of age (mice with enlarged tumors were sacrificed according to the IACUC protocol), and their anterior prostate (AP) tissues or tumors were dissected and weighed. Tissues were fixed in 10% neutral-buffered formalin (Sigma) over night and then preserved in 70% ethanol at 4°C. Fixed tissues were further processed by ethanol dehydration and embedded in paraffin according to standard protocols (Histoserv Inc., Gaithersburg, MD).

### Cell proliferation, transformation and invasion assays

Mouse embryonic fibroblasts (MEFs) were prepared from individual embryos of various genotypes at E13.5-day according to the procedure previously described [25, 26]. MEFs were cultured in Dulbecco's modified Eagle's medium (DMEM, GIBCO) supplemented with 10% fetal bovine serum (FBS), 2 mM glutamine, and Pen/Strep (100 U/ml, GIBCO) in an incubator with 5% CO_2_ at 37°C. MEFs at passage 2 were infected with retroviruses expressing Cre-PURO-IRES-GFP (pMSCV-Cre-PURO-IRES-GFP) or empty vector (pMSCV-PURO-IRES-GFP). MEFs selected with puromycin (2 μg/ml) for 2 days were used for cell proliferation, transformation and invasion assays. The cell proliferation and transformation assay were performed as previously described [26]. For migration assay, MEFs were cultured with DMEM in 60 mm Petri dishes until confluent. Wound scratching was made using sterile 200 μl tips followed with 2 washes in 1 x PBS. After cultured continuously in fresh DMEM overnight, MEFs in dishes were fixed with 10% formalin for 15 min and photographed. Wounding healing (migration) rate was determined by a ratio of grids with cells at 16 hr to grids at 0 hr.

### Bioluminescence imaging (BLI) in mice

Bioluminescence imaging (BLI) was applied to non-invasively monitor the growth of prostate tumors of *Luc^pc+^*; Pten*^pc−/−^*; Trp53*^pc−/−^* male mice. Briefly, mice anesthetized with 3% isoflurane were administered with D-Luciferin (Goldbio, MO) via I.P. at 125 mg/kg at 5 min before acquisition. Mice were then placed in the chamber of In-Vivo MS FX PRO optical imaging system (Carestream, NY), and photons were collected for a period of 1 min. The summary luminescent intensity of region of interest (ROI) was quantified using Molecular Imaging software v.5.0.7.22 (Carestream, NY). To study CRPC in mice, mutant mice were subjected to castration surgery by removing their testicles at 14 weeks of age. The regressive and/or recurrent growth of prostate tumors in castrated mice was monitored weekly by BLI for firefly luciferase activities.

### Cell culture, transfection, shRNA and mutagenesis

LNCaP, C4-2B (M.D. Anderson, TX), DU145, CWR22Rv1, PC3 (ATCC) human PCa cells were grown in RPMI 1640 medium with 10% FBS and 1% Pen/Strep in an incubator with 5% CO2 at 37°C. 293FT cells were maintained in DMEM complemented with 10% FBS and 1% Pen/Strep at 37°C with 5% CO_2_. For transient transfection, cells at 70% confluence were transfected with plasmids using Lipofectamine 2000 (Invitrogen). Expression of target genes was determined 48–72 hr post-transfection using real-time qPCR or Western blotting as described [52]. To generate SKP2 shRNA plasmids, forward and reverse oligonucleotides (Supplementary Table S1) were suspended separately in ddH_2_O and then mixed in annealing buffer (5 μl of 10 x NEB buffer 2 plus 35 μl of H_2_O). Annealed inserts were ligated into pLKO.1 TRC vector at AgeI and EcoRI sites. To knockdown SKP2 in cells, lentiviruses carrying SKP2 shRNA or scrambled sequence were prepared from 293FT cells transfected with a triple-plasmid system. Briefly, 293FT cells in 10 cm Lysine-coated petri dishes were co-transfected with 5 μg of SKP2 scrambled or SKP2 shRNA plasmids, together with 3 μg of psPAX2 packaging plasmids, and 2 μg of pMD2.G envelope plasmids using Lipofectamine 2000. Forty eight hr post-transfection, viral supernatants were collected and filtered through 0.45 μm filter. Fresh lentiviruses were applied to PC3 cells containing 5 μg/ml of Polybrene. After additional 48 hr, infected cells were selected with 2 μg/ml of puromycin for 7 days and pooled for analysis [15]. Flag-JARID1B mutants were generated from pEV-Flag-JARID1B [53] using QuikChange II XL Site-Directed Mutagenesis Kit (Agilent Technologies, CA) according to manufacturer's instruction. The nucleotides sequences of various primers for SKP2 shRNA, and JARID1B mutants are shown in Supplementary Table S1.

### Real-time reverse transcription PCR

Real-time reverse transcription PCR (real time RT-PCR) was performed according to the procedure as previously described [52]. Briefly, 5 μg of total RNA extracted from PC3-scrambled or PC3-shSKP2 cells were subjected to cDNA synthesis by reverse transcription with SuperScript III first strand synthesis kit (Invitrogen). Real-time qPCR was performed with a Bio-Rad CFX96 Real-time system in triplicate using the forward and reverse primers listed in Supplementary Table S1.

### Western blotting and half-life determination of JARID1B

Cell lysates were prepared in RIPA buffer (1×PBS, 1% Nonidet P40/Triton X-100, 0.5% sodium deoxycholate, 2mM EDTA, with or without 0.1% SDS) with protease inhibitor cocktail (Roche) followed with a brief sonication. Antibodies used were: rabbit anti-H3K4me3 (1:10,000, Cell Signaling, 9727), rabbit anti-H3 (1: 10,000, Abcam, Ab1791), rabbit anti-Skp2 (1:500, H-435, Santa Cruz, sc-7164), rabbit anti-JARID1B (1:10,000, Novus Biologicals, NB100-97821, NBP1-84352), mouse monoclonal anti-β-actin (1:10,000, Sigma, AC-74), mouse ant-Flag M2 affinity gel (Sigma, A2220), mouse anti-Flag M2 antibody (1:1000, Sigma, F1804), mouse anti-C-Myc (1:1000, Santa Cruz, sc-40), mouse anti-β-Galactosidase (1:1000, LSBio, 10B2), mouse anti-TRAF6 (1:1000, Santa Cruz, D-10), rabbit anti-TRAF6 (1:1000, Cell Signaling, D21G3). The protein stability of JARID1B in PC3 cells were performed as previously described [26]. PC3-scrambled and PC3-shSKP2 cells were incubated with 100 μg/ml of cycloheximide (CHX, Sigma) in starvation medium to inhibit further protein synthesis. To determine the stability of JARID1B proteins, cell lysates were collected at indicated time points after CHX treatment, and then subjected to Western blotting analysis using rabbit anti-JARID1B antibodies. Protein bands were quantified with Image J software. The protein degradation rate is represented as half-life (t_1/2_), which is defined as the time for 50% of the protein degraded.

### *In vivo* ubiquitination assay

*In vivo* ubiquitination assays were performed as previously described [14, 54]. Briefly, HEK293T cells were transfected with Flag tagged JARID1B [53] or JARID1B mutants, HA tagged Ubiquitin WT or Ubiquitin mutants (K48-only and K63-only) [55], Myc tagged TRAF6 [56], along with or without Myc tagged SKP2 plasmids as indicated for 24 hr, then treated with 10 μM of MG132 for additional 6 hr. Cells were lysed in 100 μl of SDS lysis buffer containing 50 mM Tris-HCl pH 7.5, 150 mM NaCl and 1% SDS, and boiled for 10 min at 95°C. Cell lysates were briefly sonicated, then diluted 10-fold with IP buffer (50 mM Tris-HCl pH 7.5, 150 mM NaCl, 1% Triton X-100, 2 mM EDTA, protease inhibitor cocktail), and immunoprecipitated with 30 μl bed volume of anti-Flag M2 affinity gel at 4 °C overnight. After the beads were washed 5 times, the precipitated proteins were eluted with 2x SDS loading buffer, and applied to Western blotting analysis with indicated antibodies.

### Immunofluorescence (IF) and immunohistochemistry (IHC)

Immunofluorescence (IF) stainings were performed as previously described [52]. Briefly, PC3-scrambled and PC3-shSKP2 cells were grown on cover slips in culture medium for 24 hr. Cells were fixed with methanol at –20°C, then incubated with indicated rabbit anti-JARID1B (1:250, Novus Biologicals, NB100-97821) and mouse anti-SKP2 (1:400, Invitrogen, 2C8D9), or mouse anti-C-Myc (1:200, Santa Cruz, 9E10), or mouse anti-Fibrillarin (1:400, Abcam, ab4566), or mouse anti-Ub-K63 (1:200, Millipore, 05-1313), or mouse anti-TRAF6 (1:100, Santa Cruz, D-10), or mouse anti-β-Galactosidase (1:100, LSBio, 10B2). IHC staining on mouse tissue sections and human PCa tissue microarray were performed as previously reported [52]. Paraffin-embedded sections of mouse tissues in 5 μm thickness were de-paraffinized in xylene for 3X 10 min, and rehydrated in graded alcohol, boiled in antigen retrieval citrate buffer, pH 6.0 for 15 min, quenched in 3% H_2_O_2_, and blocked with 10% FBS in 1 x PBS containing 0.1% Triton X-100 and 1% BSA for 1 hr. These sections were then probed with primary antibodies: rabbit anti-Skp2 (1:50, Santa Cruz, H-435), rabbit anti-JARID1B (1:250, Novus Biologicals, NB100-97821), rabbit anti-H3K4me3 (1:200, Cell Signaling, 9727), rabbit anti-TRAF6 (1:100, Cell signaling, D21G3), rabbit anti-Ki67 (Abcam 16667) for 16 hr. The sections were then stained with biotinylated secondary antibodies for 1 hr. The immune complex was visualized with ABC kit using the chromogen DAB substrate (Vector Labs). The nuclei were counterstained with Gill 3 Hematoxylin (Thermoscientific). Human prostate tissue microarray slides were purchased from Biomax, which consists of 35 cancer cases and 5 normal cases in 80 cores. Tissue sections were probed with primary antibodies: mouse anti-SKP2 (1:250, Invitrogen, 2C8D9), rabbit anti-H3K4me3 (1:200, Cell Signaling, 9727). The scores of SKP2, H3K4me3 were graded as: 0 (negative staining), 1 (weak staining), 2 (moderate staining), or 3 (strong staining) according to their staining intensities [57].

### Statistical analysis

Statistics analysis was performed using two-tail Student's *t-*test. For correlation analysis, Pearson correlation test and Chi-square test were used. The values of *P* < 0.05 were considered statistically significant.

## SUPPLEMENTARY FIGURES AND TABLES


