# Supplementary material for: SKP2 inactivation suppresses prostate tumorigenesis by mediating JARID1B ubiquitination
Source: Oncotarget. 2014 Dec 23;6(2):771–88. doi: 10.18632/oncotarget.2718 (PMC4359254; doi:10.18632/oncotarget.2718)
Supplement: Supplementary file 1 [file oncotarget-06-771-s001.pdf]

SUPPLEMENTARY FIGURES AND TABLES

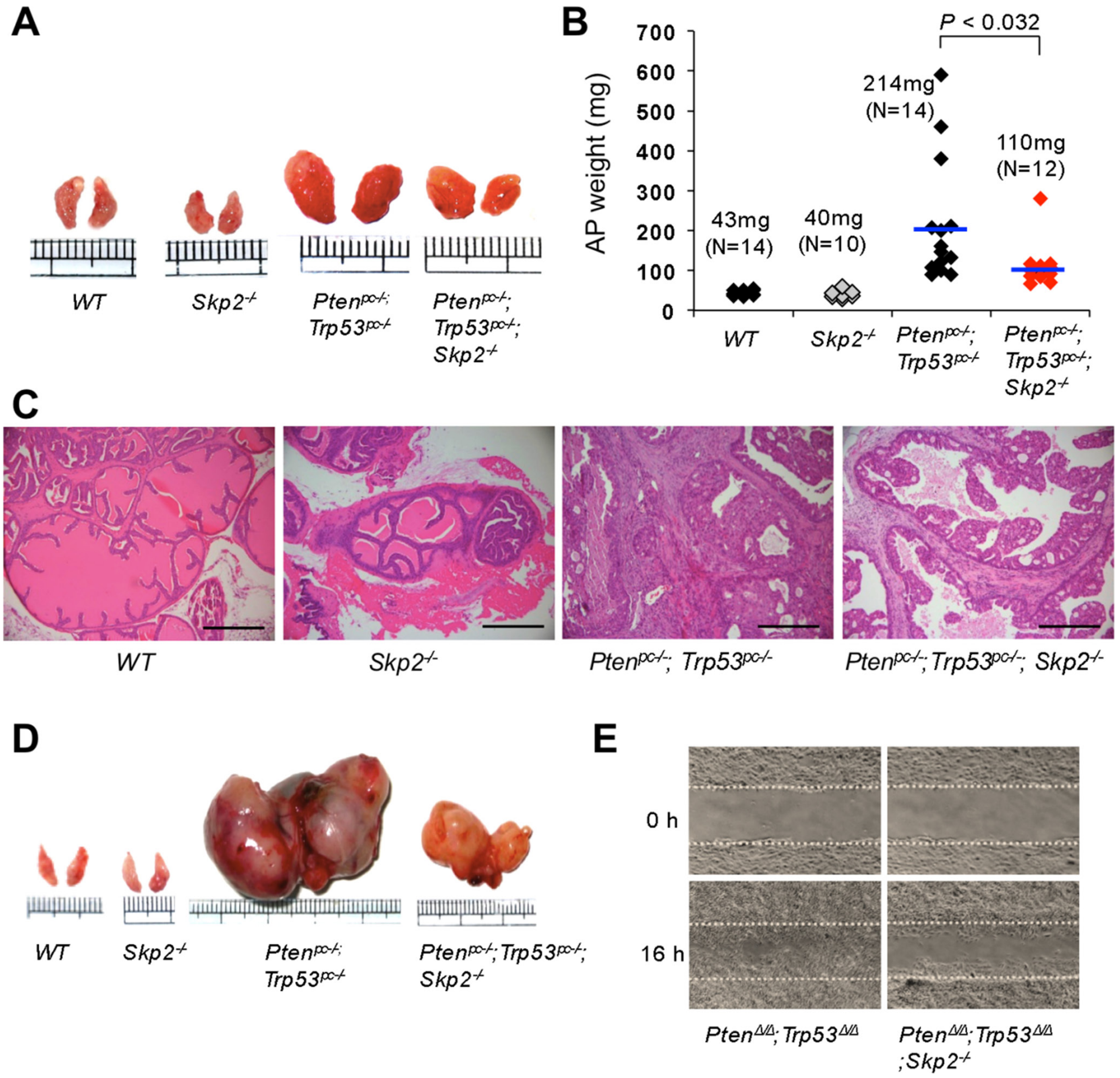

Supplementary Figure S1: Skp2 inactivation suppresses prostate tumor initiation of *Pten*$^{pc-/-}$*;Trp53*$^{pc-/-}$ mutant mice. **(A)** Biopsies of dissected anterior prostates (AP) from indicated genotypes of mice at 3 months of age. **(B)** The weight distribution of AP prostates or tumors in mice from A. N indicates the mouse number. **(C)** Representative images of H&E staining show the pathological changes of indicated genotypes of mice from A. The high-grade prostatic intraepithelial neoplasia (HG-PIN) and cancer lesions are found in *Pten*$^{pc-/-}$*;Trp53*$^{pc-/-}$ mice, while only PIN is found in age-matched *Pten*$^{pc-/-}$*;Trp53*$^{pc-/-}$*;Skp2*$^{-/-}$ mice. Scale bars represent 200 µm. **(D)** Biopsies of dissected anterior prostates (AP) from indicated genotypes of mice at 6 months of age. **(E)** Wound healing assay of Pten/Trp53 null MEFs upon Skp2 inactivation.

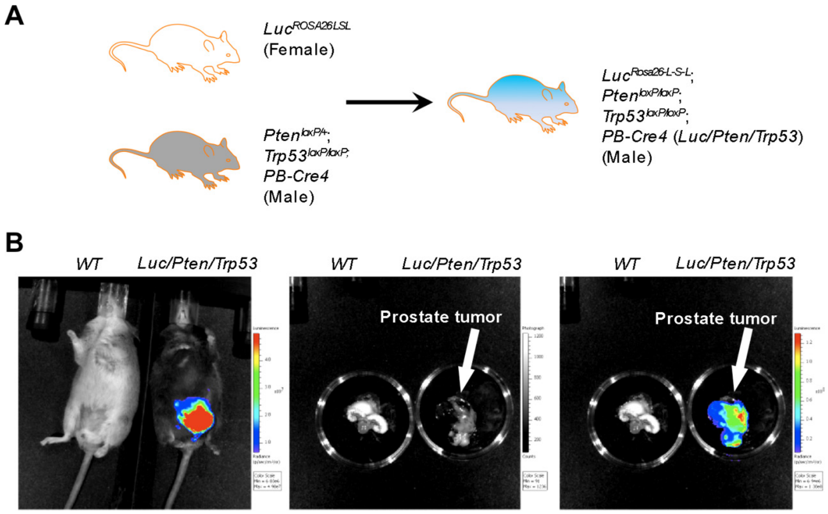

Supplementary Figure S2: Prostate tumors of *Pten/Trp53* null mutant mice are prostatic-specifically labeled with luciferase. (A) A breeding strategy to generate prostate-specific luciferase-labeled *Pten/Trp53* mutant mice. Luciferase transgenic female mice ($Luc^{ROSA26\ LSL}$) were crossed with $Pten^{loxP/+}$; $Trp53^{loxP/loxP}$ *Probasin-Cre4* male mice. Their mice in F2 offspring were further used to generate Luc^{LSL}; $Pten^{loxP/loxP}$; $Trp53^{loxP/loxP}$; *Probasin-Cre4* (referred to Luc^{pc+}; $Pten^{pc-/-}$; $Trp53^{pc-/-}$ or *Luc*/*Pten*/*Trp53*) compound mutants. **(B)** The luciferase is constrained to enlarged prostate tumors in *Luc/Pten/Trp53* mice after puberty. Left: bioluminescence images of adult *Luc/Pten/Trp53* mouse, and the control is littermate wild type mouse. Middle and Right: *Ex vivo* bright light and bioluminescence images of prostate tissues of mice from the left panel.

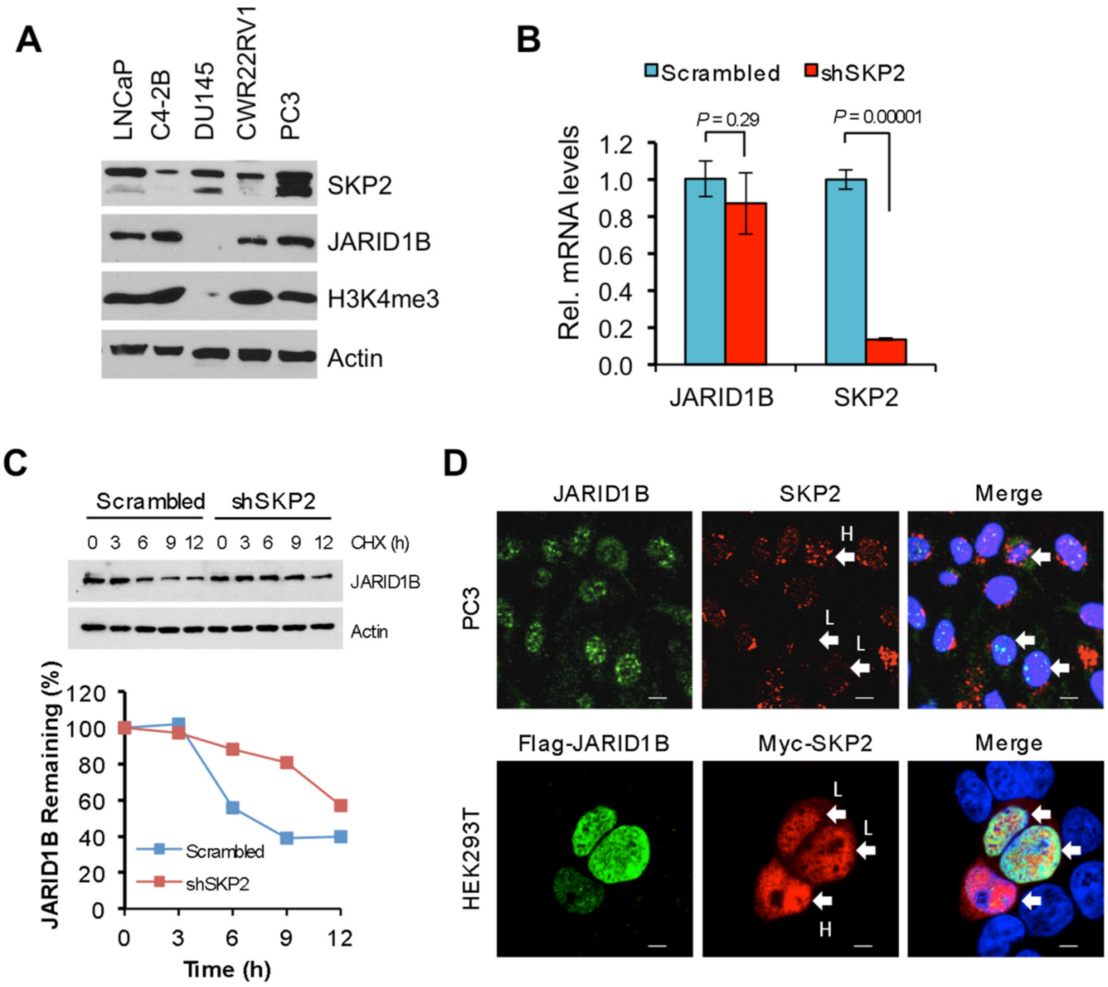

Supplementary Figure S3: SKP2 contributes to JARID1B and H3K4me3 in prostate cancer cells. (A) SKP2, JARID1B and H3K4me3 expression in human prostate cancer cell lines. **(B)** Quantitative RT-qPCR analysis to show the mRNA levels of JARID1B in PC3 cells upon SKP2 knockdown. Error bars represent means ± SD of triplicates. **(C)** SKP2 knockdown by shRNA prolongs the half-life of JARID1B. Top panel: Western blot analysis for the half-life of JARID1B upon SKP2 knockdown in PC3 cells. The inhibition of protein synthesis by 100 μg/ml of cyclohexminde (CHX) is shown at defined time points. Bottom panel: the quantification of JARID1B normalized to β-actin from Western blot in the top panel. **(D)** SKP2 reversely correlates with JARID1B in human prostate cancer cells. IF images showing a reverse correlation of expression levels of endogenous SKP2 and JARID1B proteins in PC3 cells (Top panel), and exogenously expressed Myc-SKP2 and Flag-JARID1B in HEK293T cells (Bottom panel), as indicated by arrows. H: high, L: low. The co-localizations of Myc-SKP2 and Flag-JARID1B are also shown in HEK293T cells. Scale bars represent 5 μm.

A

RAEAMNIKIEPEETTEARTHNLRRRMGCPTPKCENEKEMKSSIKQEPIERKDYIVENE
242 278 285

B

Residue	Score	Ubiquitinated
242	0.93	High confidence
278	0.93	High confidence
285	0.97	High confidence

Label	Score range	Sensitivity	Specificity
Low confidence	0.62 ≤ s ≤ 0.69	0.464	0.903
Medium confidence	0.69 ≤ s ≤ 0.84	0.346	0.950
High confidence	0.84 ≤ s ≤ 1.00	0.197	0.989

Supplementary Figure S4: Potential ubiquitination sites on JARID1B. The primary amino acid sequence of JARID1B/KDM5B (NP_006609.3) was input into the UbPred program (http://www.ubpred.org/) [1], and the predicted ubiquitination sites were returned. **(A)** Lysine 242, lysine 278 and lysine 285 are predicated as the most probable ubiquitination sites of JARID1B by the UbPred program with a score more than 0.93 each. **(B)** Output of the scores of residues at potential ubiquitination sites of JARID1B by the UbPred software. Bottom: definition of cutoff scores for the residue of target protein.

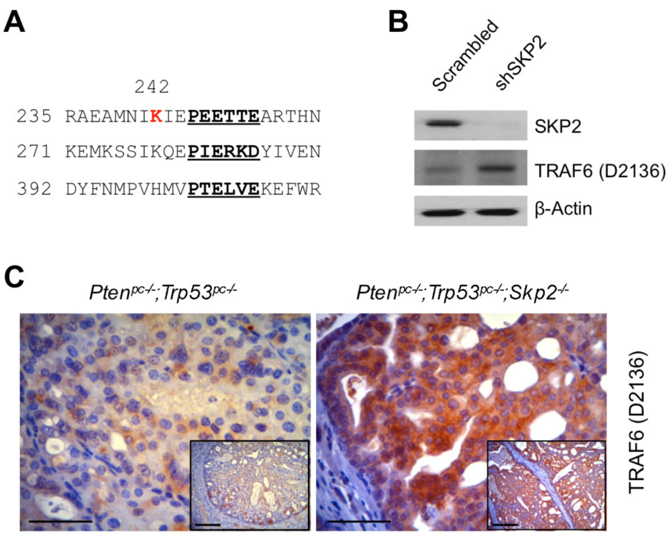

Supplementary Figure S5: SKP2 regulates TRAF6 in prostate cancer cells *in vitro* and in mice *in vivo*. **(A)** Three potential consensus PxExxAr/Ac TRAF6 binding motifs in human JARID1B as underscored. Ar is for aromatic residues, and Ac is acidic residues. The ubiquitination site lysine residue 242 in red color is adjacent to these TRAF6 binding motifs. **(B)** SKP2 knockdown elevates the levels of endogenous TRAF6 protein in PC3 cells. **(C)** Skp2 deficiency results in an elevation of TRAF6 protein in *Pten*$^{pc-/-}$;*Trp53*$^{pc-/-}$ mice as compared to the control. Scale Bars represent 50 μm.

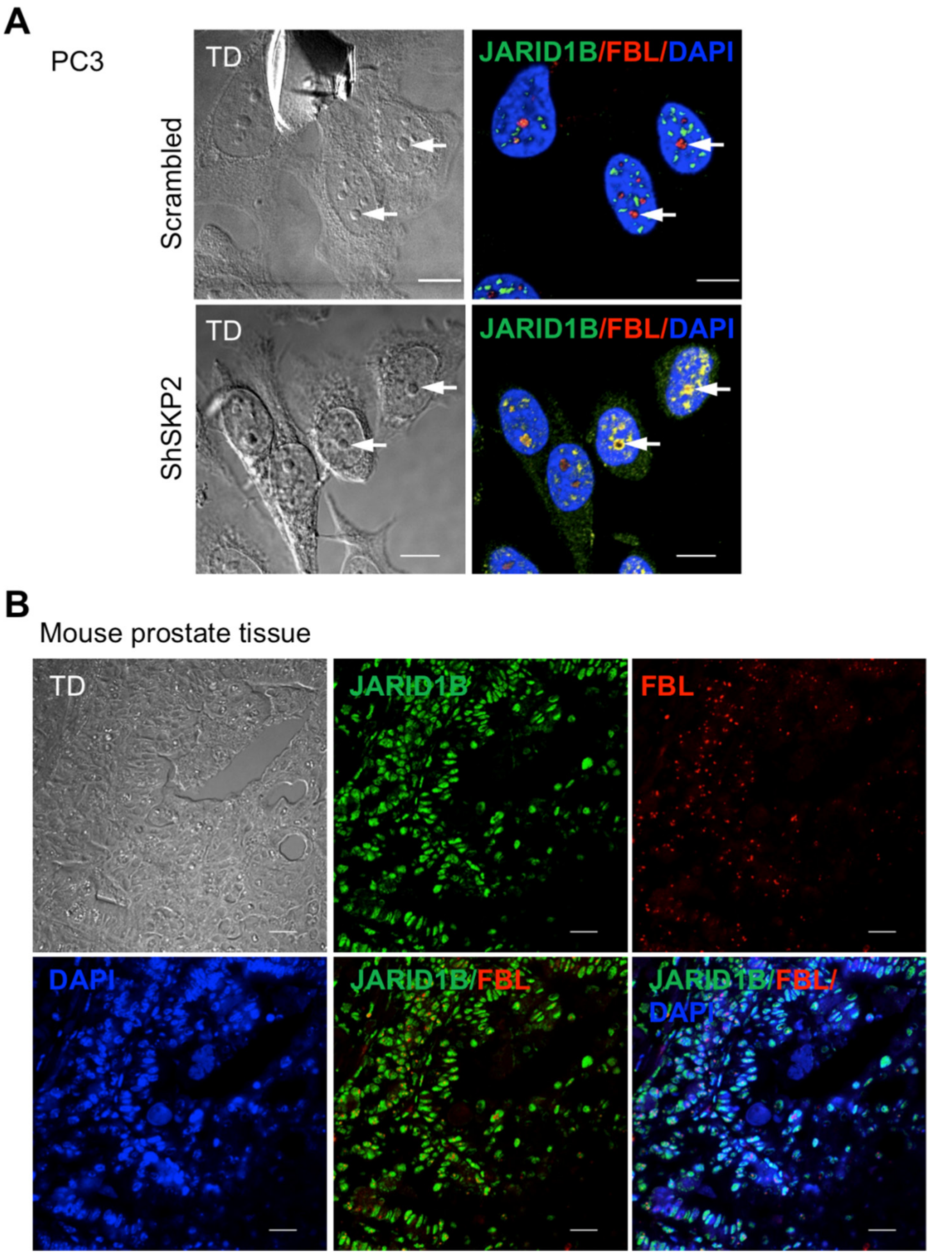

Supplementary Figure S6: JARID1B is accumulated in nucleoli of PC3 cells upon SKP2 inactivation. (A) JARID1B and Fibrillarin co-localize in the nucleoli of human prostate cancer PC3 cells upon SKP2 knockdown (Relating to Figure 5A). The white arrows indicate positions of the nucleoli. Scale Bars represent 10 µm. **(B)** Low magnification images to show the co-localization of JARID1B and Fibrillarin proteins in the nuceloli of most prostate cells from *Pten*$^{pc-/-}$*;Trp53*$^{pc-/-}$*;Skp2*$^{-/-}$ mice. FBL indicates the nucleolar marker Fibrillarin. Scale Bars represent 20 µm.

A

Four NoLSs are predicted in human JARID1B protein (NP_006609.3):

PSETCPPARRAKRMRAEAMN (between positions 221 and 240)

IVENEKEKPKSRSKKATNAVDLY (between positions 288 and 310)

GLLGLKRKQRKLKEPLPNGKKKST (between positions 1097 and 1120)

LSSERWERVKKMRTPKKKKIKLSHPKDMNNFKLE (between positions 1419 and 1452)

B.

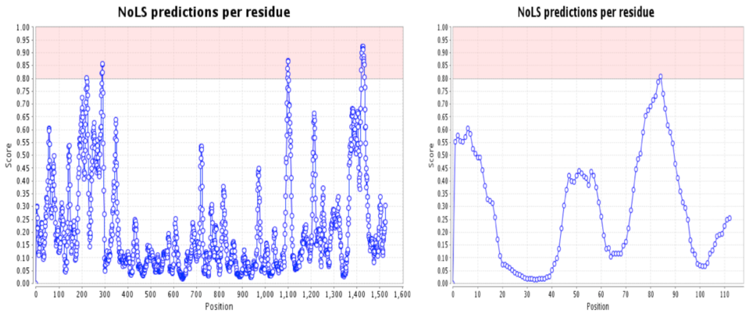

JARID1B (KDM5B) p14ARF

Supplementary Figure S7: Potential nucleolar localization sequences (NoLS) of JARID1B. The primary amino acid sequence of JARID1B/KDM5B (NP_006609.3) was input into the NOD (Nulcleolar Localization Sequence Detector) program (http://www.compbio.dundee.ac.uk/www-nod/) [2,3], the predicted NoLS sequences of JARID1B were obtained. **(A)** Four potential NoLS sequences of JARID1B predicted by the NOD program. **(B)** NoLS score per residue graphs of JARID1B and p14ARF. Known nucleolar protein p14ARF (NP_478102.2) was used for a validation of the NOD program. As expected, the program successfully predicted the genuine NoLS signal sequence of human p14ARF (AQLRRPRHSHPTRARRCPGG between positions 84 and 103).

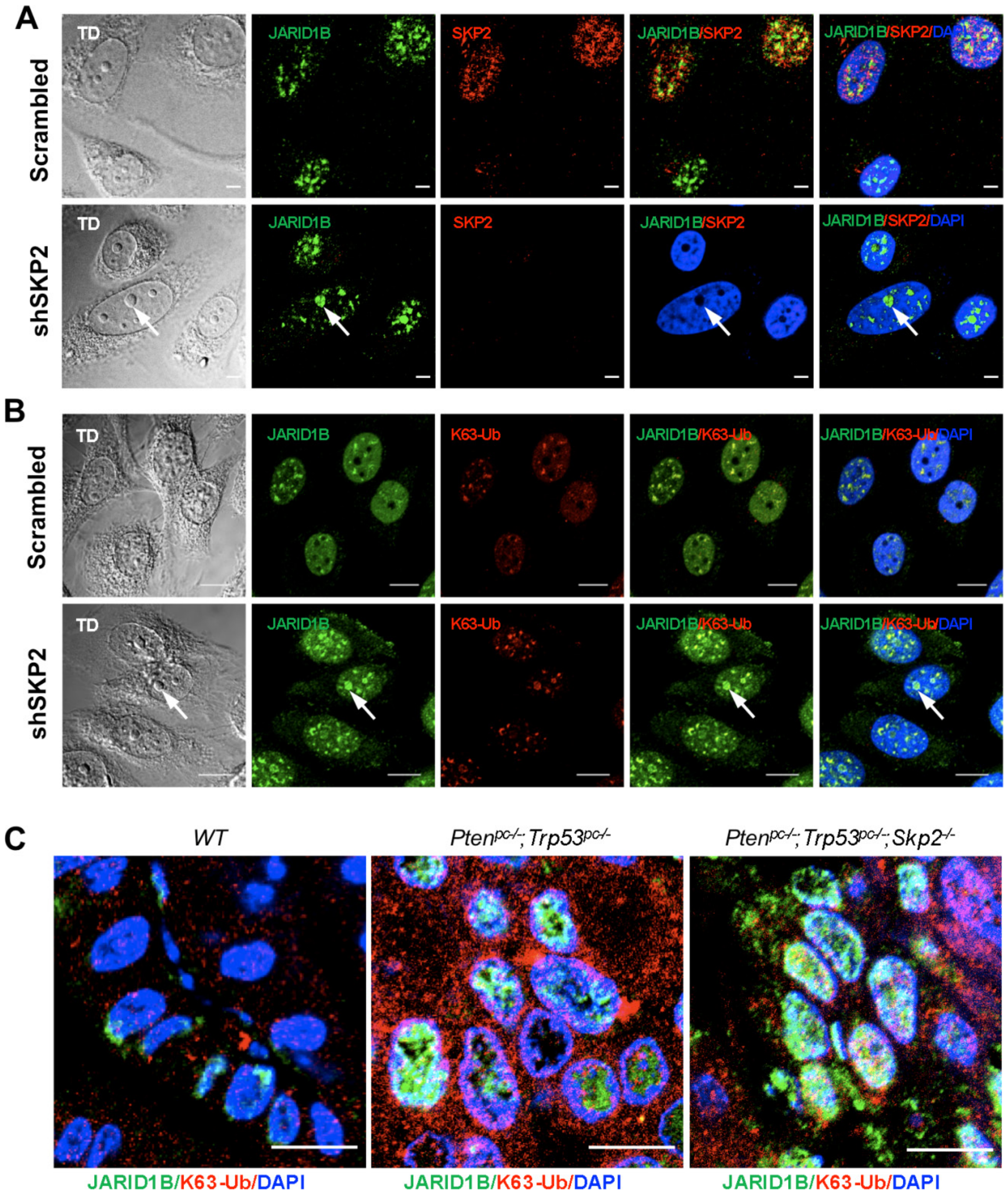

Supplementary Figure S8: Lysine 63-linked ubiquitinaiton of JARID1B determines its nucelolar localization. (A) SKP2 knockdown promotes the nucleolar localization of endogenous JARID1B in PC3 cells as indicated by arrows. Scale bars represent 5 µm. **(B)** Lysine 63-linked ubiquitination of endogenous JARID1B is associated to its nucleolar localization in SKP2 knockdown PC3 cells (Relating to Figure 5B). Scale bars represent 10 µm. **(C)** Lysine 63-linked ubiquitination of JARID1B in prostate tumors of *Pten/Trp53/Skp2* mutant mice. Scale bars represent 10 µm.

Supplementary Table S1: Genotyping PCR, Real time RT-PCR and shRNA primer sequences

Genotyping Primer name	Primer sequence	Ref.
Lpten-1	TGTTTTTGACCAATTAAAGTAGGCTGTG	[4]
Lpten-2	AAAAGTTCCCCTGCTGATGATTTGT	[4]
Lpten-3	TTCTCTTGAGCACTGTTTCACAGGC	[4]
Lp53-1	GAGACGCTGAGTCCGGTTCCCTCC	[4]
Lp53-2	GCAAGAGGTGACTTTGGGGTGAAGCTC	[4]
Cre-1	TGATGGACATGTTCAGGGATC	[4]
Cre-2	CAGCCACCAGCTTGCATGA	[4]
Skp2 WT-1	AGAGTGGAAGAACCCAGGCAGGAC	[5]
Skp2 WT-2	CCCGTGGAGGGAAAAAGAGGGACG	[5]
Skp2 KO-1	GCATCGCCTTCTATCGCCTTCTTG	[5]
Skp2 KO-2	TTCCCACCCCCACATCCAGTCATT	[5]
Luc Forward	CGG TAT CGT AGA GTC GAG GCC	[6]
L-LucB	CAG GGC GTA TCT CTT CAT AGC C	[6]
L-S-L-LucB	GGC CAG AGG CCA CTT GTG TAG C	[6]
**qRT-PCR primer name**		
Actin-mRNA-F	CTGAAGTACCCCATCGAGCACG GCA	[7]
Actin-mRNA-R	GGATAGCACAGCCTGGA TAGCAACG	[7]
JARID1B-mRNA-F	TTCCACAGCTTGCTGAGATG	[8]
JARID1B-mRNA-R	GCCATAGCTTTCTCCACTGC	[8]
SKP2-mRNA-F	CCCACGGAAACGGCTGAAGA	[9]
SKP2-mRNA-R	CGCTAGGCGATACCACCTCTTACAA	[9]
**Skp2 shRNA primer name**		
Skp2-shRNA1-S	CCGGGCCTAAGCTAAATCGAGAGAACTCGAGTT CTCTCGATTTAGCTTAGGCTTTTTG	[10]
Skp2-shRNA1-AS	AATTCAAAAAGCCTAAGCTAAATCGAGAGAACT CGAGTTCTCTCGATTTAGCTTAGGC	[10]
Skp2-shRNA2-S	CCGGGATAGTGTCATGCTAAAGAATCTCGAGATT CTTTAGCATGACACTATCTTTTTG	[5]
Skp2-shRNA2-AS	AATTCAAAAAGATAGTGTCATGCTAAAGAATCTC GAGATTCTTTAGCATGACACTATC	[5]
Skp2-scramble-S	CCGGATGAGTCAACGCGAATACAGACTCGAGTC TGTATTCGCGTTGACTCATTTTTTG	
Skp2-scramble-AS	AATTCAAAAAATGAGTCAACGCGAATACAGACT CGAGTCTGTATTCGCGTTGACTCAT	
LKO 5’	TGGACTATCATATGCTTACCGTAAC	
LKO 3’	GTATGTCTGTTGCTATTATGTCTA	

(*Continued*)

Primer name	Primer sequence	Ref.
**Flag-JARID1B mutants primer name**		
pEV-JARID1B-K242R-F	GAGCAGAGGCCATGAATATTAGGATAGAACCCGAGGAGACAACGG	
pEV-JARID1B-K242R-R	CCGTTGTCTCCTCGGGTTCTATCCTAATATTCATGGCCTCTGCTC	
pEV-JARID1B-K285R-F	GCATCAAGCAAGAACCTATTGAGAGGAGGGAT TATATTGTAGAAAATGAGAAGG	
pEV-JARID1B-K285R-R	CCTTCTCATTTTCTACAATATAATCCCTCCTCTCAATA GGTTCTTGCTTGATGC	

Supplementary Table S2: Statistical analysis of the correlation between SKP2 and H3K4me3 in human prostate microarray tissues by immunohistochemistry. The statistical differences were determined by Chi-Square test. The expression levels of SKP2 and H3K4me3 were graded with intensity score 0 (negatively stained), 1 (weakly stained), 2 (moderately stained), and 3 (strongly stained), as described previously [11]

H3K4me3 detection level (Intensity score)	SKP2 detection level (Intensity score)				
		**0**	**1**	**2**	**3**
	**0**	10	0	1	0
	**1**	4	9	0	0
	**2**	2	2	4	2
	**3**	1	0	0	0
	Degrees of freedom: 9; Chi: 29.07; *P*-value: 0.0006				

SUPPLEMENTARY REFERENCES

1. Radivojac P, Vacic V, Haynes C, Cocklin RR, Mohan A, Heyen JW, Goebl MG, Iakoucheva LM. Identification, analysis, and prediction of protein ubiquitination sites. Proteins. 2010; 78:365–380.
2. Scott MS, Boisvert FM, McDowall MD, Lamond AI, Barton GJ. Characterization and prediction of protein nucleolar localization sequences. Nucleic Acids Res. 2010; 38:7388–7399.
3. Scott MS, Troshin PV, Barton GJ. NoD: a Nucleolar localization sequence detector for eukaryotic and viral proteins. BMC Bioinformatics. 2011; 12:317.
4. Chen Z, Trotman LC, Shaffer D, Lin HK, Dotan ZA, Niki M, Koutcher JA, Scher HI, Ludwig T, Gerald W, Cordon-Cardo C, Pandolfi PP. Crucial role of p53-dependent cellular senescence in suppression of Pten-deficient tumorigenesis. Nature. 2005; 436:725–730.
5. Lin HK, Chen Z, Wang G, Nardella C, Lee SW, Chan CH, Yang WL, Wang J, Egia A, Nakayama KI, Cordon-Cardo C, Teruya-Feldstein J, Pandolfi PP. Skp2 targeting suppresses tumorigenesis by Arf-p53-independent cellular senescence. Nature. 2010; 464:374–379.
6. Safran M, Kim WY, Kung AL, Horner JW, DePinho RA, Kaelin WG Jr. Mouse reporter strain for noninvasive bioluminescent imaging of cells that have undergone Cre-mediated recombination. Mol Imaging. 2003; 2:297–302.
7. Hoppe BL, Conti-Tronconi BM, Horton RM. Gel-loading dyes compatible with PCR. Biotechniques. 1992; 12:679–680.
8. Mitra D, Das PM, Huynh FC, Jones FE. Jumonji/ARID1 B (JARID1B) protein promotes breast tumor cell cycle progression through epigenetic repression of microRNA let-7e. J Biol Chem. 2011; 286:40531–40535.
9. Bhatt KV, Hu R, Spofford LS, Aplin AE. Mutant B-RAF signaling and cyclin D1 regulate Cks1/S-phase kinase-associated protein 2-mediated degradation of p27Kip1 in human melanoma cells. Oncogene. 2007; 26:1056–1066.
10. Wu J, Lee SW, Zhang X, Han F, Kwan SY, Yuan X, Yang WL, Jeong YS, Rezaeian AH, Gao Y, Zeng YX, Lin HK. Foxo3a transcription factor is a negative regulator of Skp2 and Skp2 SCF complex. Oncogene. 2013; 32:78–85.
11. Lakshmikanthan V, Zou L, Kim JI, Michal A, Nie Z, Messias NC, Benovic JL, Daaka Y. Identification of beta-Arrestin2 as a corepressor of androgen receptor signaling in prostate cancer. Proc Natl Acad Sci U S A. 2009; 106:9379–9384.
